# Donor–Acceptor Complexes of (5,10,15,20-Tetra(4-methylphenyl)porphyrinato)cobalt(II) with Fullerenes C_60_: Self-Assembly, Spectral, Electrochemical and Photophysical Properties

**DOI:** 10.3390/molecules27248900

**Published:** 2022-12-14

**Authors:** Nataliya G. Bichan, Ekaterina N. Ovchenkova, Varvara A. Mozgova, Alexander A. Ksenofontov, Nadezhda O. Kudryakova, Ivan V. Shelaev, Fedor E. Gostev, Tatyana N. Lomova

**Affiliations:** 1 G.A. Krestov Institute of Solution Chemistry of the Russian Academy of Sciences, Akademicheskaya Str. 1, 153045 Ivanovo, Russia; 2 N.N. Semenov Federal Research Center for Chemical Physics Russian Academy of Sciences, Kosigin Str. 4, 119991 Moscow, Russia

**Keywords:** cobalt(II)porphyrin, fullerene, fullero[60]pyrrolidine, noncovalent bonds, supramolecular architectures with well-defined geometry, bonding kinetics, spectroscopy, redox properties, photoinduced electron transfer

## Abstract

The noncovalent interactions of (5,10,15,20-tetra(4-methylphenyl)porphinato)cobalt(II) (CoTTP) with C_60_ and 1-N-methyl-2-(pyridin-4-yl)-3,4-fullero[60]pyrrolidine (PyC_60_) were studied in toluene using absorption and fluorescence titration methods. The self-assembly in the 2:1 complexes (the triads) (C_60_)_2_CoTTP and (PyC_60_)_2_CoTTP was established. The bonding constants for (C_60_)_2_CoTTP and (PyC_60_)_2_CoTTP are defined to be (3.47 ± 0.69) × 10^9^ and (1.47 ± 0.28) × 10^10^ M^−2^, respectively. ^1^H NMR, IR spectroscopy, thermogravimetric analysis and cyclic voltammetry data have provided very good support in favor of efficient complex formation in the ground state between fullerenes and CoTTP. PyC_60_/C_60_ fluorescence quenching in the PyC_60_/C_60_–CoTTP systems was studied and the fluorescence lifetime with various CoTTP additions was determined. The singlet oxygen quantum yield was determined for PyC_60_ and the intensity decrease in the ^1^O_2_ phosphorescence for C_60_ and PyC_60_ with the CoTTP addition leading to the low efficiency of intercombination conversion for the formation of the ^3^C_60_* triplet excited state was found. Using femtosecond transient absorption measurements in toluene, the photoinduced electron transfer from the CoTTP in the excited singlet state to fullerene moiety was established. Quantum chemical calculations were used for the determination of molecular structure, stability and the HOMO/LUMO energy levels of the triads as well as to predict the localization of frontier orbitals in the triads.

## 1. Introduction

Among the wide variety of fullerenes, C_60_ is one of the most common and actively studied fullerenes due to its pronounced electron-withdrawing property and great potential for application in many areas of applied science [1,2,3,4,5,6]. C_60_ fullerene is known to be a strong singlet oxygen generator, and it has only limited use in photodynamic therapy due to its extremely low solubility in water [7]. However, there are investigations devoted to the study of the effect of aqueous suspensions of C_60_ fullerene on microorganisms [8,9]. The modification of the fullerene core by attaching various groups or introducing a metal atom into the carbon cage provides the possibility of fine-tuning the physical and chemical properties of these compounds. The fullerene derivatization increases its solubility in an aqueous solvent and reduces aggregation to possible application in medicinal chemistry as antibacterial/antifungal agents [10,11,12,13]. The transition to trifluoromethyl derivatives of C_60_ leads to the increase in the electron affinity compared to initial fullerene and in the ability to form long-lived anion-radical particles [14,15]. The dimerization of fullerenes, namely the formation of pyrrolizidine/cyclobutane-bridged double-caged fullerene derivatives, promotes the charge delocalization over both carbon cages, upshifts energies of frontier molecular orbitals, a narrowed bandgap and a reduced electron-transfer reorganization energy relative to molecular C_60_ [16,17]. The endohedral C_60_ derivatives are interesting because a metal atom encapsulated into C_60_ is able to transfer a part of its spin density to the carbon cage, which ensures long spin relaxation times [18]. The modification of C_60_ by pyridyl groups, when the pyridyl nitrogen atom donates its lone pair of electrons to the electron-deficient π-system of fullerene, is used in the control of electron-withdrawing properties (LUMO energy) of fullerene derivatives. Such modification also allows for the design of various donor–acceptor systems, providing the high efficiency of photoinduced separation of charges when irradiated with the visible light [19,20,21,22,23].

One of the strategies for improving the charge-separation process is the formation of covalent and noncovalent supramolecular systems based on fullerenes and electro/photoactive compounds as acceptors and donors, respectively [24]. Porphyrins and their complexes are promising among such compounds due to their structural diversity, photophysical/photochemical properties, broadband spectrum, high extinction coefficients, stability at relatively high temperatures and the presence of a π-conjugated macrocycle [25,26]. These systems offer new opportunities in the preparation of materials that may produce long-lived charge-separated states in high quantum yields.

The supramolecular interactions of the fullerenes with the porphyrins have been extensively studied in recent decades [27,28]. However, there is little data on the interaction of the non-functionalized fullerene C_60_ with metalloporphyrins (MPs). The C_60_ complexes with Zn(II), Co(II) and Fe(III) octaethylporphyrins (OEPs) bonding due to the van der Waals interactions were obtained by M.M. Olmstead et al. in 1999 [29]. Their structures, established by X-ray diffraction, contained one C_60_ molecule surrounded by two metalloporphyrin molecules in the case of ZnOEP and CoOEP, but one metalloporphyrin molecule in the case of ClFeOEP [29]. In the work of [30], molecular complexes of fullerene C_60_ with Mn(II), Co(II), Cu(II), Zn(II) and Fe(III) tetraphenylporphyrins (TPPs) were synthesized and studied by ESR, IR, UV–vis and X-ray photoelectron spectroscopy. The formation of molecular complexes with fullerenes affects the ESR spectra of MPs lowering the spin state in the case of MnTPP or changing the constant of hyperfine interaction in the case of CoTPP and CuTPP [30]. Additionally, C_60_-containing complexes based on jaw-like Pd, Zn, Cu, Co, Fe and Mn bis(metalloporphyrins) were described in the work of [31]. In 2007, Aida et al. reported that iridium(III) porphyrin cyclic dimer bonds C_60_ to form the 1:1 complex [32]. In 2017, Yamada et al. have synthesized iridium porphyrin bearing peripheral alkyl chains, which forms not only the 1:1 complex but also the 2:1 one in toluene [33]. The electronic interactions between zinc(II) porphyrin containing the reactive amino groups and C_60_ were established by steady state fluorescence, time correlated single photon counting measurements and DFT calculations [34]. These new data point to the consideration of metalloporphyrin–C_60_ systems as the basis for numerous photovoltaic devices.

The self-assembly of the cobalt(II) porphyrin (CoP) coordination triads (where the triad is the molecule with various combinations of electron donor and acceptor) with fullero[60]pyrrolidines have been reported in our early works [35,36,37,38], while the one with non-functionalized fullerene C_60_ have not been explored to date. We report here about the ability of cobalt(II) porphyrin bearing peripheral 4-methylphenyl groups to bond both C_60_ and PyC_60_, thus forming the 2:1 coordination complexes. The self-assembly in cobalt(II) porphyrin–C_60_/PyC_60_ systems in toluene was studied by the absorption and fluorescence titration methods. The spectral properties of triads in the ground and excited states are observed and discussed in this article.

## 2. Results and Discussion

The UV–vis and fluorescence spectroscopy are suitable methods to study the supramolecular interaction of metalloporphyrins upon bonding fullerenes [39,40,41]. Both methods allow one to easily determine the stability constants of the CoTTP–C_60_/PyC_60_ triads. The spectrophotometric methods are attractive due to the large changes in the CoPs UV–vis spectra when a non-covalent bond is formed along the axial axis causes.

**Figure 1:**
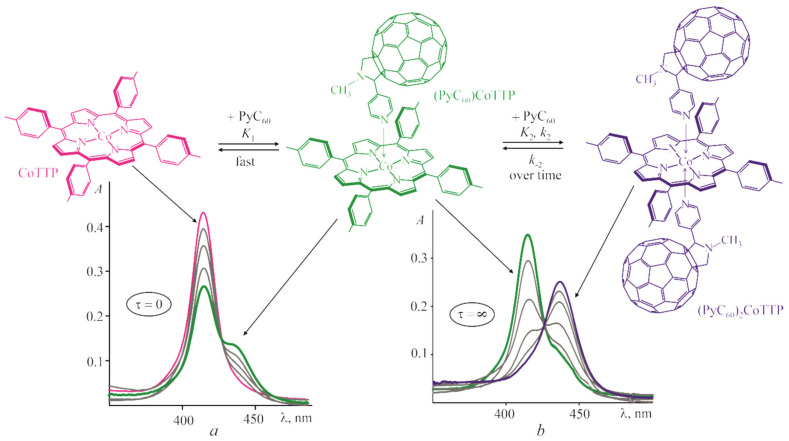
The scheme of the (PyC_60_)_2_CoTTP formation and UV–vis spectrum transformations during the titration of CoTTP with PyC_60_ (C_PyC60_ = 0 ÷ 7.7 × 10^−5^ M) in toluene immediately after mixing the reactants (**a**) and at the end of the reactions (**b**).

Figure 1 shows the scheme of the CoTTP reaction with PyC_60_ and the corresponding UV–vis spectrum transformations during the titration of the CoTTP solution in toluene by PyC_60_. The time-dependent titration at τ = 0 and τ = ∞ [42] allowed us to describe both rapid/slow equilibriums and the rate of the second-stage reaction. The fast reversible (PyC_60_)CoTTP formation (at τ = 0) is characterized by the gradual decrease in the intensity at 414 nm in the CoTTP spectrum. The (PyC_60_)_2_CoTTP formation occurs over time (within 100–1100 s depending on the PyC_60_ concentration, see Figure S1 in Supplementary Materials). It is accompanied by the gradual decrease in the absorption at 414 nm together with the appearance of the new band at 437 nm. The analysis of the log*I*-log*C*_PyC60_ dependence slope has confirmed one PyC_60_ molecule bonding at the first and second stage (Figure S2, Supplementary Materials). The step constants, *K*_1_ and *K*_2_, were calculated by the equation for the three-component equilibrium system (see Equation (S1) in Supplementary Materials) and were found to be (2.3 ± 0.3) × 10^4^ and (6.4 ± 0.9) × 10^5^ M^−1^, respectively. The stability constant of the (PyC_60_)_2_CoTTP triad, β = *K*_1_ × *K*_2_ is (1.47 ± 0.28) ×10^10^ M^−2^.

The rate of forward reactions (*k*_2obs_) in the slow equilibrium was measured (Table S1, Supplementary Materials). Using the data in Table S1 and Figure S3, the first order with respect to CoTTP and PyC_60_, respectively, are determined (Figure S3, Supplementary Materials). The rate constant *k*_2_ found from Figure S3 data is (62.3 ± 5.1) M s^−1^. The rate constant *k*_−2_ equal *k*_2_/*K*_2_ is 9.7 × 10^−5^ M^2^ s^−1^.

**Figure 2:**
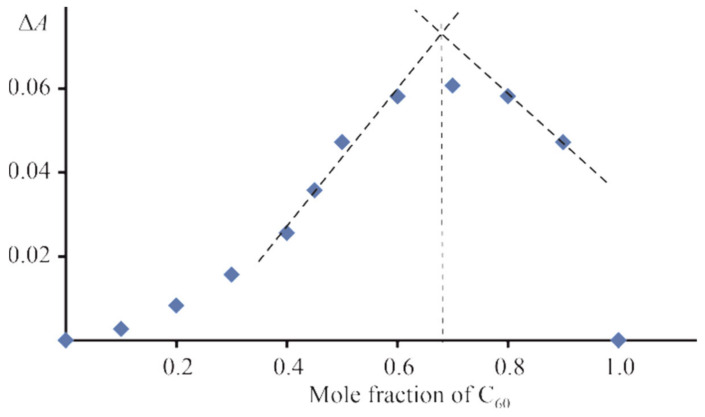
Job’s plot for CoTTP–C_60_ system in toluene; in Job’s experiment, the concentration of CoTTP and C_60_ are continuously varied in the concentration range from 1.06 × 10^−6^ M to 1.06 × 10^−5^ M.

UV–vis spectral changes observed during CoTTP bonding with C_60_ are similar to the ones for the CoTTP–PyC_60_ system. The formation of a supramolecular complex between CoTTP and C_60_ is accompanied by the Soret band shift from 414 nm to 432 nm. Job’s plot points to the CoTTP–C_60_ bonding stoichiometry of 1:2 (Figure 2). The use the time-dependent titration method for the CoTTP–C_60_ system allowed us to also identify two stages (the fast equilibrium and slow two-way reaction of C_60_ coordination, Figure S4, Supplementary Materials) and to determine the thermodynamic stability constants, which were found to be *K*_1_ = (5.02 ± 0.94) × 10^4^ M^−1^, *K*_2_ = (6.63 ± 0.98) × 10^4^ M^−1^ and β = (3.47 ± 0.69) × 10^9^ M^−2^.

**Figure 3:**
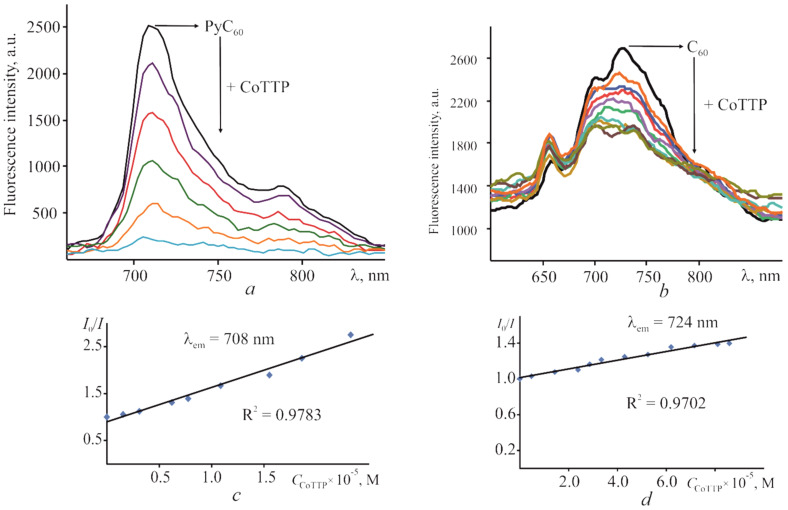
The changes in the PyC_60_ stationary fluorescence spectrum (*C*_PyC60_ = 4.39 × 10^−6^ M) in the presence of CoTTP (*C*_CoTTP_ = 1.55 × 10^−6^–2.33 × 10^−5^ M) (**a**) and the ones for C_60_ (*C*_C60_ = 9.08 × 10^−5^ M) in the presence of CoTTP (*C*_CoTTP_ = 4.77 × 10^−6^–8.58 × 10^−5^ M) (**b**) in toluene; (**c**) Stern–Volmer plot for the PyC_60_–CoTTP (**c**)/C_60_–CoTTP (**d**) system in toluene. λ_exc_ = 450 nm.

The stability constants (the formation ones) of (PyC_60_)_2_CoTTP and (C_60_)_2_CoTTP were also determined with the help of steady state fluorescence measurements in toluene. Since cobalt(II) porphyrins were non-fluorescent [43], and C_60_ [44] as well as their derivatives [45] were fluorescent with a low fluorescence quantum yield (3.2 × 10^−4^ for C_60_), we studied the fluorescence of C_60_/PyC_60_ with various additions of CoTTP at thermostating solutions for 20 min (τ = ∞). The fluorescence quantum yield of PyC_60_ determined in the toluene is 5.3 × 10^−4^. It was established that the C_60_/PyC_60_ fluorescence upon excitation at 450 nm is quenched following the gradual addition of CoTTP. The steady state fluorescence titration experiments for the PyC_60_–CoTTP and C_60_–CoTTP systems recorded in toluene are demonstrated in Figure 3a,b, respectively. The process of C_60_/PyC_60_ fluorescence quenching with CoTTP was investigated using the Stern–Volmer equation. The results are given in Figure 3c,d. The Stern–Volmer plot is linear in both cases. The fluorescence-quenching constants (*K*_SV_) were found to be (5.47 ± 1.34) × 10^4^ and (5.66 ± 0.45) × 10^3^ M^−1^ for (PyC_60_)_2_CoTTP and (C_60_)_2_CoTTP, respectively. The stability constants (*K*_BH_) for the triads were determined by the Benesi–Hildebrand equation plotting *I*_0_/(*I*_0_ − *I*) vs. 1/[C_CoTTP_] (Figure S5, Supplementary Materials), the straight line was obtained and the value of *K*_BH_ was determined as (1.2 ± 0.3) × 10^10^ and (1.3 ± 0.3) × 10^9^ M^−1^ for (PyC_60_)_2_CoTTP and (C_60_)_2_CoTTP, respectively. These values are in agreement with the values from UV–vis spectrophotometric titration, although the standard deviation in the latter case is approximately 1.3 times less.

According to the literature data [46], the fluorescence lifetime of C_60_ varies from 1.17 to 1.3 ns. As the results of our measurements, the fluorescence lifetime of C_60_ in toluene was determined to be 1.38 ns. C_60_ exhibits bi-exponential decay which is in agreement with the works of [47,48]. PyC_60_ also exhibits bi-exponential decay with the fluorescence lifetime of 1.61 ns.

The time-resolved fluorescence spectroscopy results for the C_60_, PyC_60_, C_60_–CoTTP and PyC_60_–CoTTP solutions in toluene are shown in Table S2, Supplementary Materials. The different effect in the C_60_ and PyC_60_ fluorescence lifetime from the CoTTP addition points to the different nature of fullerene quenching. The C_60_ fluorescence lifetime decreases to 1 ns with the addition of 2.4 × 10^−5^ M CoTTP. The PyC_60_ fullerene fluorescence lifetime remains unchanged in the PyC_60_–CoTTP mixtures. This behavior is associated with the static and dynamic mechanism of fluorescence quenching upon bonding CoTTP with PyC_60_ and C_60_, respectively.

**Figure 4:**
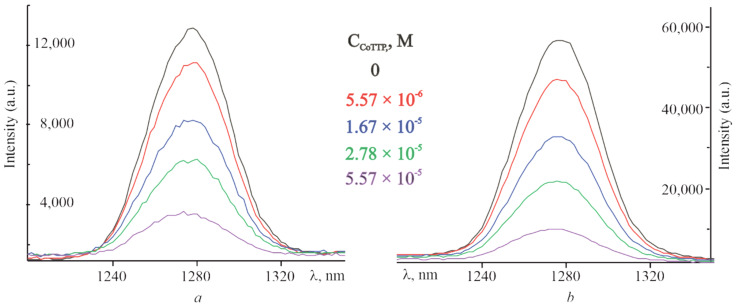
The emission spectra of singlet oxygen formed by the sensitization with C_60_ (**a**), PyC_60_ (**b**), C_60_–CoTTP (**a**) and PyC_60_–CoTTP (**b**).

Another feature of fullerenes is their ability to generate singlet molecular oxygen (^1^O_2_) with high quantum yields [49], which we have used to confirm the formation of supramolecular systems with the charge-separation state [50]. The ^1^O_2_ quantum yield for C_60_ is often equated to 1 [51,52]. Given these data, the quantum yield of singlet oxygen of PyC_60_ was determined and found to be 0.7 (see Section 3.4). This value is consistent with the higher triplet energies and the lower quantum yields of triplet and singlet oxygen for functionalized C_60_ [53]. The intensity of the ^1^O_2_ phosphorescence decreases when the addition of CoTTP is increased. Emission spectra of the ^1^O_2_ obtained by the photoirradiation (λ = 453 nm) of the C_60_, PyC_60_, C_60_–CoTTP and PyC_60_–CoTTP solutions are shown in Figure 4. The phosphorescence decay of singlet oxygen formed by the sensitization with PyC_60_ and PyC_60_–CoTTP is represented in Table S3, Supplementary Materials. The intensity of the ^1^O_2_ phosphorescence for C_60_ and PyC_60_ decreases with the maximum CoTTP addition by 3.5 and almost 6 times, respectively. This fact additionally confirms the realization of photoinduced electron transfer (PET) in (PyC_60_)_2_CoTTP/(C_60_)_2_CoTTP from the donor CoTTP fragment to the acceptor fullerene-containing fragment, since this process possibly leads to the low efficiency of intercombination conversion for the formation of the ^3^C_60_* triplet excited state.

**Figure 5:**
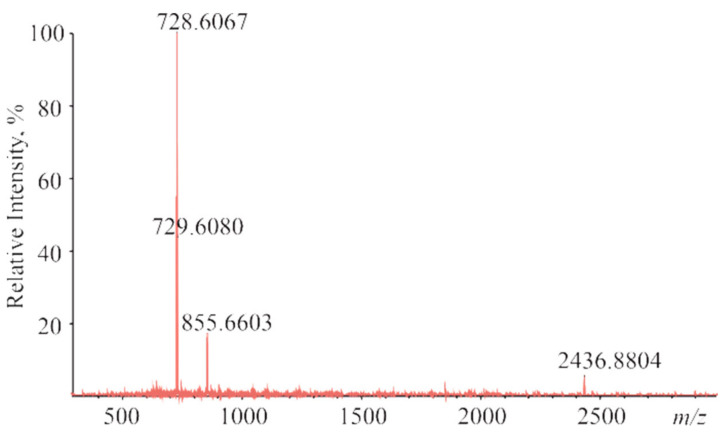
The MALDI-TOF mass spectrum of (PyC_60_)_2_CoTTP.

The interactions between CoTTP and C_60_/PyC_60_ in the ground states are confirmed by spectral methods such as mass spectrometry and IR/^1^H NMR spectroscopy. MALDI mass spectrometry is a rapid qualitative test for fullerene bonding by porphyrin [31]. Using the dithronol matrix, (PyC_60_)_2_CoTTP gives the exact mass peaks corresponding to both the supramolecular complex (2436.88 [M]^+^) and its constituents (728.61 [CoTTP]^+^ and 855.66 [PyC_60_]^+^) (Figure 5). No molecular ion peak of (C_60_)_2_CoTTP was observed due to the dissociation of the noncovalent complex in the conditions of the mass spectral experiments.

**Figure 6:**
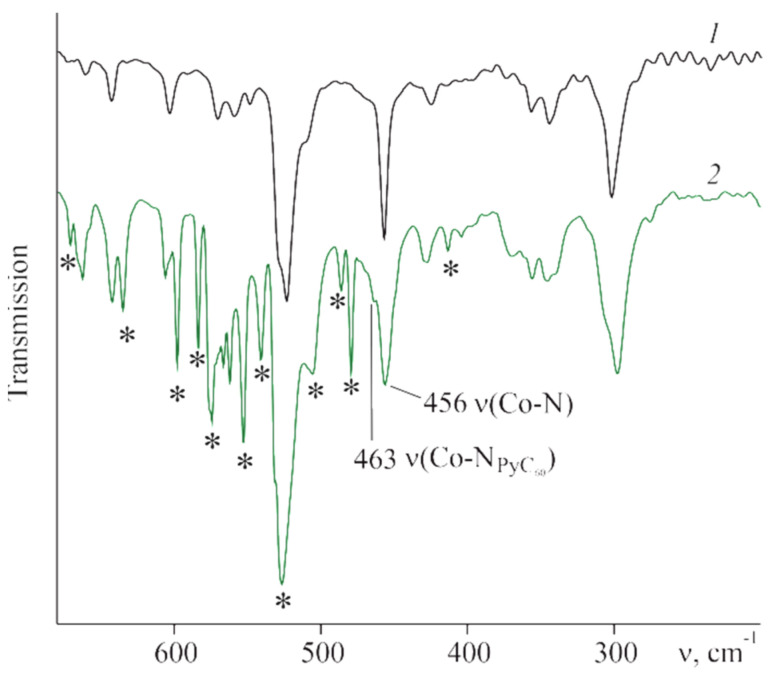
The IR spectra of CoTTP (*1*) and (PyC_60_)_2_CoTTP (2) in CsBr. The peaks corresponding to vibrations of PyC_60_ are denoted with asterisks.

According to IR spectral studies, the position and intensity of the pyrrole ring stretching vibrations of CoTTP remain practically unchanged in the IR spectra of (PyC_60_)_2_CoTTP and (C_60_)_2_CoTTP, pointing to the Co atom location in the macrocycle plane [54,55]. This is related to the signals at 1684 cm^−1^, ν (C=N), 1452 cm^−1^, 1404 cm^−1^, 1377 cm^−1^ ν(C-H)_pyr_ in the CoTTP spectrum that is listed in Experimental Section. The planar coordination center was also confirmed by both theoretical calculations (see next section) and the data from the work of [42] on the example of (2,3,7,8,12,18-hexamethyl,13,17-diethyl,5-(2-pyridyl)porphinato)cobalt(II) and its triads with PyC_60_. Along with the signals of the porphyrin macrocycle, there are signals of coordinated PyC_60_ in the IR spectrum of (PyC_60_)_2_CoTTP (Table S4, Supplementary Materials). The vibration signals of the C_60_ skeleton at 527 cm^−1^, 574 cm^−1^ and 1428 cm^−1^ as well as signals of pyridyl/pyrrolidinyl fragments of the fullerene core at 1245 cm^−1^, 1281 cm^−1^ and in the 400–710 cm^−1^ range (Figure 6, Table S4, Supplementary Materials) are observed. These signals are shifted by 1–14 cm^−1^ relative to uncoordinated PyC_60_ [36,42]. The signal at 456 cm^−1^ related to ν(Co-N) is considerably broadened; the ν(Co-N_PyC60_) signal appears at 463 cm^−1^ as the shoulder (Figure 6). The IR (C_60_)_2_CoTTP spectrum is the sum of the C_60_ [56] and CoTTP spectra, suggesting the weak interaction between C_60_ and CoTTP in the ground state.

**Figure 7:**
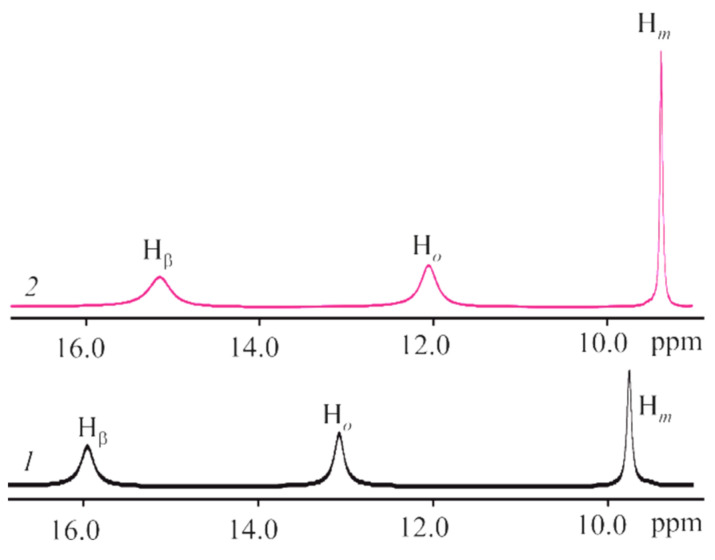
The ^1^H NMR spectra of CoTTP (*1*) and its triad, (PyC_60_)_2_CoTTP (*2*) in CDCl_3_.

The CoTTP ^1^H NMR spectrum (Figure S6, Supplementary Materials), typical of other cobalt(II) porphyrins [35,36,37], is represented by the signals of β-pyrrole, ortho-phenyl and meta-phenyl protons as the broadened singlets at 15.98 ppm, 13.07 ppm and 9.75 ppm, respectively. The upfield shifts of these proton signals in the ^1^H NMR spectrum of the (PyC_60_)_2_CoTTP (Figure 7) due to the ring current effect of the fullerene on the porphyrin indicate the fullerene coordination state.

The thermal stability of (PyC_60_)_2_CoTTP/(C_60_)_2_CoTTP studied in comparison with the its precursors by the TGA method demonstrates the high parameters of most porphyrins [57,58]. Figure S7a, Supplementary Materials, shows the TG curve and its first derivative (DTG) for CoTTP. The weight loss of 0.85% between 25 °C and 479 °C is connected with the desorption of solvent molecules rather than the metalloporphyrin degradation. The elimination of the tolyl groups and the degradation of macrocycle ring manifest itself in the form of the weight loss of 12% in the temperature range of 479–509 °C with the major weight loss at 504 °C and of 19.65% between 719 °C and 750 °C with the maximum value at 738 °C, respectively. C_60_ has also displayed the high thermal stability, exhibiting the decomposition process in the range of 827–937 °C (Figure S7b, Supplementary Materials). The TGA results for PyC_60_ (Figure S7c, Supplementary Materials) show the start of the compound decomposition from around 374 °C to 454 °C, i.e., at a considerably lower temperature than the one for pure C_60_. The main drop in the PyC_60_ mass corresponding to the fullerene cage decomposition occurs between 807 °C and 904 °C; the peak of the derivative on the weight loss is observed at 883 °C.

**Figure 8:**
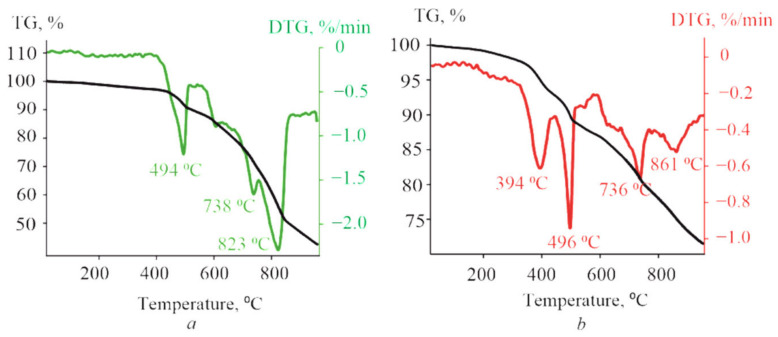
The TG and DTG curves for the (C_60_)_2_CoTTP (**a**) and (PyC_60_)_2_CoTTP (**b**) powder from 25 °C to 920 °C. The heating rate is 10 °C/min.

The decomposition of triads shown in Figure 8 is the multi-stage process which includes the degradation of both the porphyrin macrocycle and the fullerene core. In the case of (C_60_)_2_CoTTP, the peaks of the derivative on the weight loss at 494 °C/738 °C and 823 °C correspond to the thermal decomposition of CoTTP and C_60_, respectively (Figure 8a). The thermal behavior of (PyC_60_)_2_CoTTP (Figure 8b) was similar. The peaks of the derivatives on the weight loss for both triads are observed at temperatures lower than that of their individual components. Thus, the results of the TGA experiment suggest the direct bonding between CoTTP and C_60_/PyC_60_ in the high stable triads.

**Figure 9:**
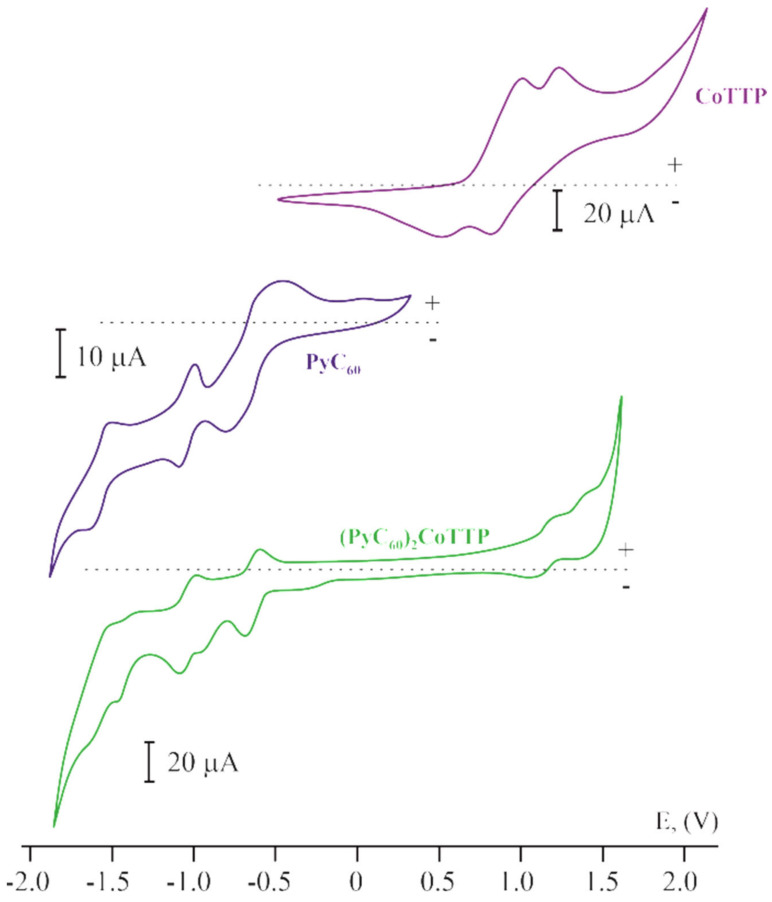
The cyclic voltammograms for CoTTP, PyC_60_ and (PyC_60_)_2_CoTTP in CH_2_Cl_2_ containing the 0.1 M (n-Bu)_4_NClO_4_ supporting electrolyte. The scan rate is 100 mV s^−1^.

The cyclic voltammograms (CVs) of the triads and their individual components are represented in Figure 9. There are two reversible peaks in the positive potential range for CoTTP. The first and the second oxidation/reduction correspond to the central metal atom and the macrocycle, respectively [59]. The cyclic voltammograms of C_60_ and PyC_60_ in CH_2_Cl_2_ are characterized by three pairs of peaks, which is typical of fulleropyrrolidines [60,61]. The redox potentials for all these compounds are given in Table S5, Supplementary Materials. The CV of (C_60_)_2_CoTTP and (PyC_60_)_2_CoTTP are essentially the sum of the CoTTP and C_60_/PyC_60_ peaks. Three well-pronounced reversible redox waves corresponding to the sequential addition of electrons to the fullerene unit are observed in the negative potential window. Two reversible redox waves in the positive potential window correspond to the central metal atom and the macrocycle of CoTTP. However, these peak potentials are shifted relative to ones for the individual components (Table S5, Supplementary Materials), which indicates the interaction between the π system of CoTTP and C_60_/PyC_60_ and the redistribution of the electron density within the triad.

**Figure 10:**
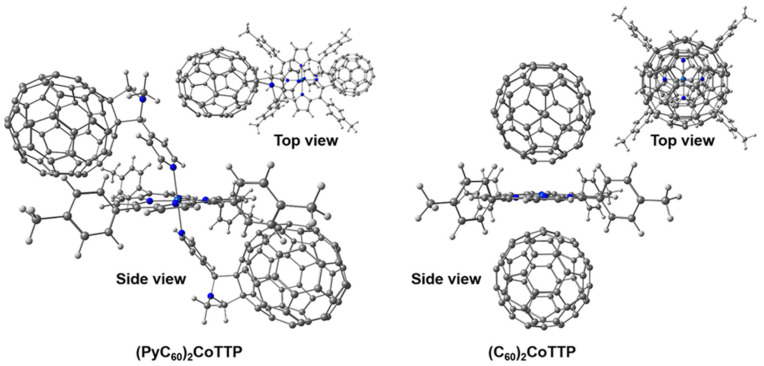
The optimized structures of (PyC_60_)_2_CoTTP (**left**) and (C_60_)_2_CoTTP (**right**).

The DFT calculations have demonstrated the higher stability of (PyC_60_)_2_CoTTP compared with (C_60_)_2_CoTTP. The bonding energy in (PyC_60_)_2_CoTTP and (C_60_)_2_CoTTP calculated as the difference between the total energy of triad minus the sum of the energies of CoTTP and C_60_/PyC_60_ is 253 kJ·mol^−1^ and 293 kJ·mol^−1^, respectively. The coordination of CoTTP is accompanied by a distortion of the macrocycle and its deviation from planarity by 7.31° (Figure 10). The coordination center has an octahedral geometry with average values of Co–N_macrocycle_ and Co–N_PyC60_ bond lengths of 2.002 Å and 2.382 Å, respectively (Figure S8a, Supplementary Materials). The additional (PyC_60_)_2_CoTTP stabilization is facilitated by both π-π CoTTP–PyC_60_ stacking and the peripheral tolyl CH_3_–PyC_60_·π interactions.

In contrast to donor–acceptor bonding, the effect of CoTTP–C_60_ π-π stacking in the macrocycle geometry, with a slight distortion of 1.47°, is softer. The average length of the Co–N_macrocycle_ bonds is 2.004 Å (Figure S8b, Supplementary Materials); the distance between the centers of the CoTTP and C_60_ closest contacts is 2.686 Å. In addition, one can note the presence of the peripheral tolyl CH_3_—C_60_·π contacts. The characteristic feature of the considered triads is the charge transfer from the porphyrin macrocycle to the acceptor fullerene moieties. This is clearly seen as the decrease in negative charge of the CoTTP nitrogen atoms in (PyC_60_)_2_CoTTP and (C_60_)_2_CoTTP (Figure S9, Supplementary Materials). At the same time, the pyrrolidine moiety in (PyC_60_)_2_CoTTP and the fullerene fragment in (C_60_)_2_CoTTP acts as the donors (the latter due to the interactions of the carbon atoms of the electron-saturated [6,6] double bonds with the complexing agent atom), transferring the electron density to the metal atom. This is confirmed by the CoTTP central atom positive charge decrease in (PyC_60_)_2_CoTTP and (C_60_)_2_CoTTP (Figure S9, Supplementary Materials). The axial coordination does not lead to the cobalt atom displacement from the macrocycle plane.

**Figure 11:**
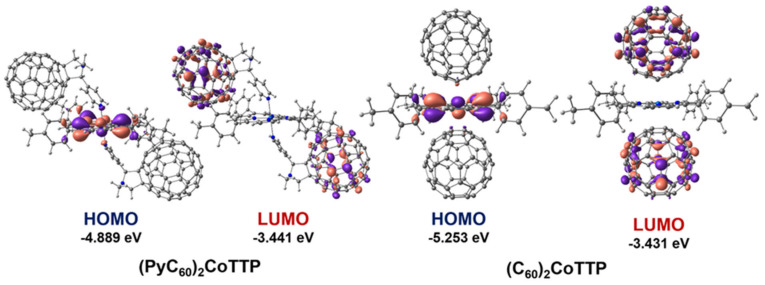
The FMOs in (PyC_60_)_2_CoTTP (**left**) and (C_60_)_2_CoTTP (**right**).

The analysis of the frontier molecular orbitals (FMOs) of (PyC_60_)_2_CoTTP and (C_60_)_2_CoTTP has shown that HOMO and LUMO in (PyC_60_)_2_CoTTP are localized on CoTTP/the pyridine moiety nitrogen atom and on both PyC_60_ fullerene moieties, respectively (Figure 11). In (C_60_)_2_CoTTP, HOMO is localized on both CoTTP and the carbon atoms of the electron-saturated [6,6] double bonds while LUMO is localized on both C_60_ moieties (Figure 11). Such localization of FMOs in the triads may indicate the mechanism of the implementation PET from the donor CoTTP moiety to the acceptor fullerene-containing moiety. The triad HOMO–LUMO gaps obtained from the DFT calculations (Figure 11) are consistent with the ones estimated from the oxidation and reduction potentials as difference between the first CoTTP oxidation and the first fullerene reduction potentials [60]. The HOMO–LUMO gap is 1.19 V and 1.45 V for (C_60_)_2_CoTTP and (PyC_60_)_2_CoTTP, respectively.

The information about the excited state of CoTTP and its linear spectra dynamic obtained by the transient absorption measurements using a femtosecond pump–supercontinuum probe setup is as follows: as shown in Section 3.2, the CoTTP Soret band at 414 nm displays an almost 15 times higher molar extinction coefficient than the Q band at 529 nm. The negative signal at 416 nm related to ground-state bleaching and dominating the spectral range of transient absorption spectra was excluded from the studied spectral window for greater visibility and greater significance of the positive signals of in femtosecond transient absorption (FTA) spectra. The CoTTP FTA spectra recorded after the 435 nm laser pulse excitation were collected between 430 nm and 780 nm in different time windows (Figure S10, Supplementary Materials). The CoTTP FTA spectrum displays the characteristic spectral shape of porphyrin singlet excited states [39]. There is the broad intense absorption band with the maximum at 443 nm as well as the Q-band at 527 nm in the CoTTP singlet excited state spectrum, which bleaches at the respective ground-state absorption band (Figure S10, Supplementary Materials). The excitation wavelength 435 nm used in the present work corresponds to the transition from S0 to the vibrationally excited level of the S2 state of porphyrins [32]. The analysis of transient absorbance kinetics at 443 nm at short delays has revealed the fast rise component with a time constant of only 0.11 ps, which can be presumably attributed to the S2→S1 internal conversion (Figure S11, Supplementary Materials). Thus, this time constant can be interpreted as the lifetime of the S2 excited state (τ_1_). This is in agreement with the data reported earlier for other Co(II) porphyrins [19,55]. The τ_1_ values of 0.08 ps, 0.17 ps and 0.099 ps were found for the toluene solutions of (5,10,15,20-tetraphenylporphinato)cobalt(II), (5,10,15,20-tetra(4-trifluoromethylphenyl)porphinato)cobalt(II) [62] and meso-carbazole substituted cobalt(II)porphyrins, respectively [26]. The singlet excited state (S1) lifetime (τ_2_) of CoTTP was calculated from the monoexponential fit to the decay profile at 443 nm and was found to be 5.95 ps (Figure S11, Supplementary Materials).

**Figure 12:**
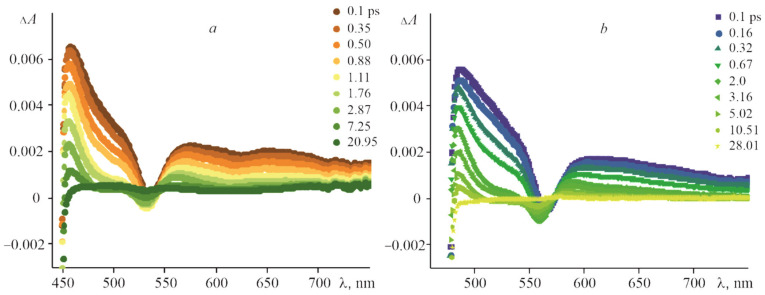
The FTA spectra at various time delays for (PyC_60_)_2_CoTTP (**a**) and (C_60_)_2_CoTTP (**b**) in toluene following the 435 nm laser excitation.

The (PyC_60_)_2_CoTTP and (C_60_)_2_CoTTP FTA spectra (Figure 12) differ from these for CoTTP. The detection of both the CoTTP radical cation, CoTTP^•+^ and the PyC_60_/C_60_ radical anion, PyC_60_^•−^/C_60_^•−^, would confirm PET from porphyrin to fullerene. The PyC_60_^•−^/C_60_^•−^ band is known to appear as broad near-IR peak in the 950 nm to 1020 range [43]. For the spectral range of our instrument setup from 400 nm to 850 nm, it is not possible to detect a similar peak. That is why the charge-separated states formed in the triads are confirmed in our work by the changing CoTTP^•+^ bands and S2 and S1 excited state lifetimes.

**Figure 13:**
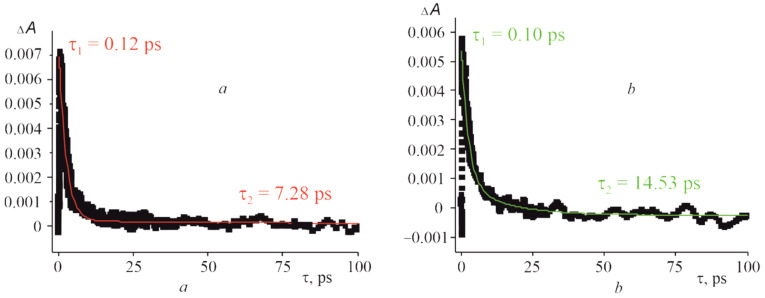
The time profile of CoTTP^•+^ band at 456 nm for CoTTP^•+^:PyC_60_^•−^ (**a**) and at 486 nm for CoTTP^•+^:C_60_^•−^ (**b**) (λ_exc_ = 435 nm) in toluene. (Red and green monoexponential fit the decay profile).

As shown in Figure 12, the positive signal at 456 nm shifted by 13 nm compared to that of CoTTP is observed after the excitation of the (PyC_60_)_2_CoTTP. This signal, displayed at 486 nm in the case of (C_60_)_2_CoTTP, shifts much more (Figure 12). The time profiles of the CoTTP^•+^ bands are represented in Figure 13. The difference within the same time observation window (100 ps) can be seen between the two triads studied. The time constants of the (PyC_60_)_2_CoTTP and (C_60_)_2_CoTTP singlet excited state, τ_2_, extracted from the CoTTP^•+^ decay are 7.28 ps and 14.53 ps, respectively. The analysis of the τ_2_ values shows a not too long lifetime for the radical ion-pairs of the triads, CoTTP^•+^:PyC_60_^•−^/C_60_^•−^, in toluene; however, these results indicate PET from CoTTP to bonded PyC_60_/C_60_. The excited state lifetime can be changed by the variation of solvent properties. The charge-separated state lifetime of triad based on CoTTP and phenyl imidazole functionalized fullero[60]pyrrolidine was found at about 1.61 ns in o-dichlorobenzene [43].

## 3. Materials and Methods

### 3.1. General Information

Organic solvents, chromatographic materials and fullerene (C_60_) were obtained from Aldrich Chemicals and Acros Organics and used as received without further purification. Meso-tetra(4-tolylphenyl)porphyrin was obtained from PorphyChem.

The UV–vis spectra were measured on an Agilent 8454 UV–visible spectrophotometer in toluene. The stationary fluorescence, emission spectra of singlet molecular oxygen and time-resolved fluorescence measurements were carried out by means of a high-performance fluorescence lifetime and steady-state spectrometer FluoTime 300 with a laser 450 nm, LDH-P-C-450, (PicoQuant, Berlin, Germany) as an excitation source. ^1^H NMR spectra were recorded on a Bruker Avance III- 500 NMR spectrometer in CDCl_3_ at room temperature. Chemical shifts (δ) were referenced to the residual solvent peak (CDCl_3_, 7.26 ppm). IR spectra were obtained using a VERTEX 80v spectrometer in KBr and CsBr pellets. The solid samples of the triads for the IR measurements were prepared by the vacuum removal of the solvent. Mass spectra were performed on an Axima Confidence (Shimadzu Biotech, a 337 nm nitrogen laser) spectrometer. Mass spectra were accumulated using reflectron positive ion mode for MALDI-TOF MS.

Thermal analysis (TG/DTG) of the complexes was carried out in argon using a Netzsch TG 209 F1 microthermal balance. The rate of sample heating, the sample weights and the temperature range were 10 °C/min, 3.5–4.7 mg and 25–920 °C, respectively. The resolution of microthermal balances is 1 × 10^−4^ mg.

The experiments of cyclic voltammetry (dichloromethane, 0.1 M (n-Bu)_4_NClO_4_) were performed on a Solartron SI 1260 using the three-electrode system (a platinum button, a platinum wire and a saturated calomel electrode (SCE) as the working, counter and reference electrode, respectively). Cyclic voltammograms were measured at the potential sweep rate of 1000 mV·s^−1^. All solutions were purged from the dioxygen prior to electrochemical measurements using an argon gas.

### 3.2. Synthesis and Analysis of Compounds

**(5,10,15,20-tetra(4-methylphenyl)porphinato)cobalt(II)**, CoTTP, was synthesized by reaction of 5,10,15,20-tetra(4-methylphenyl)porphin (30 mg, 0.041 mmol) with Co(AcO)_2_·4H_2_O (51 mg, 0.21 mmol) in boiling dimethylformamide (DMF) for 40 min. The contents of the reaction flask were cooled and diluted with water. The products were extracted into chloroform. The solution in CHCl_3_ was repeatedly washed with distilled water to remove DMF; then, CHCl_3_ was partially distilled. The residual solution was purified by chromatography on the Al_2_O_3_ column (the grade II activity according to Brockman) using chloroform. The yield is 82%. UV–vis (toluene) λ_max_/nm (logε): 414 (5.28), 529 (3.86). IR (KBr, cm^−1^) ν_max_: 3021 ν(C-H)_Ph_; 2919, 2856 ν(CH_3_); 1684 ν (C=N)_Pyr_; 1612, 1549, 1508 ν(C=C); 1452, 1404, 1377 ν(C-H)_Pyr_; 1351, 1310, 1238 ν(C–N); 1209, 1182, 1108, 1074, 1036, 1002, 967, 849, 841 δ(C-H)_Ph/Pyr_; 799, 717 γ(C-H)_Ph/Pyr_. IR (CsBr, cm^−1^) ν_max_: 661, 642, 603, 570, 558, 523, 456 ν(Co-N), 424, 356, 344, 301. ^1^H NMR (CDCl_3_), δ (ppm): 15.95 (s, 8H_β_), 13.07 (br. s, 8H_o_), 9.75 (br. s, 8H_m_), 4.16 (s, 12H_tolyl_). MS (MALDI-TOF): *m*/*z* calcd for C_48_H_36_N_4_Co [M]^+^ 727.78; found: 728.32 [M]^+^ (Figure S12, Supplementary Materials).

**1-Methyl-2-(pyridin-4′-yl)-3,4-fullero[60]pyrrolidine**, PyC_60_. The synthesis and spectral characteristics of PyC_60_ are represented in the work of [63].

### 3.3. Thermodynamics and Kinetics

The two-way reaction of CoTTP with C_60_ and PyC_60_ in toluene was spectrophotometrically studied at 298 K using the time-dependent titration method in which the thermodynamic and kinetic reaction parameters are obtained in one experimental series (see Supplementary Materials for details).

### 3.4. Fluorescence Spectroscopy

The fluorescence spectra of C_60_/PyC_60_ and its complexes with CoTTP (after excitation 450 nm (LDH-P-C-450)) were measured in toluene in quartz cuvettes (10 mm × 10 mm) on FluoTime 300 PicoQuant spectrofluorimeter. The self-assembly in the systems CoTTP–C_60_/PyC_60_ were studied using the method of the molar ratios. The molar series of the solutions with constant concentrations of C_60_/PyC_60_ and varying concentrations of CoTTP were prepared by adding CoTTP solution to the C_60_ (6.51 × 10^−5^ M) and PyC_60_ (6.55 × 10^−5^ M) solutions in toluene. To evaluate the efficiency of the C_60_/PyC_60_ fluorescence quenching, the Stern–Volmer constants (*K*_SV_) were determined using the equation:

$$I/I_{0}=1+K_{\mathrm{SV}}\cdot C_{\mathrm{CoTTP}}$$

where *I*_0_ and *I* are the fluorescence intensity of C_60_/PyC_60_ in the absence and presence of CoTTP, respectively. 

The CoTTP–C_60_/PyC_60_ bonding constants (*K*_BH_) were estimated using modified Benesi–Hildebrand equation:

$$\left(I_{max}-I_{0}\right)/\left(I_{x}-I_{0}\right)=1+\left(1/K_{\mathrm{BH}}\right)\times \left(1/C_{{\mathrm{CoTTP}}^{n}}\right)$$

where *I*_0_, *I*_x_ and *I*_max_ are the fluorescence intensities of C_60_/PyC_60_ in the absence of CoTTP, at an intermediate C_60_/PyC_60_ concentration and at the concentration of complete interaction, respectively.

The PyC_60_ fluorescence quantum yield was determined in the toluene at room temperature using C_60_ in toluene as a fluorescence standard (*Φ*_st_, ${{}_{C}}_{60}$ = 3.2×10^−4^) [44]. The value of *Φ*_F_ was calculated using the equation:

$$\Phi_{F}=\Phi_{std}\cdot \left(\frac{S_{PyC_{60}}}{S_{std}}\right)\cdot \left(\frac{A_{std}}{A_{PyC_{60}}}\right)\cdot {\left(\frac{n_{PyC_{60}}}{n_{std}}\right)}^{2}$$

where *Φ*_F_ is the fluorescence quantum yield, *Φ*_std_—the fluorescence quantum yield of the standard, *S*—the integrated fluorescence intensity (the area under spectrum), *A*—the absorbance at the excitation wavelength and *n*—the refractive index of the solvents used.

The fluorescence decay curves were measured and the fluorescence lifetimes were obtained by the reconvolution of the decay curves using the EasyTau 2 software package (PicoQuant). The biexponential fluorescence decay model was applied for C_60_, PyC_60_ and C_60_–CoTTP/PyC_60_–CoTTP systems in toluene. The instrument response function (IRF) of the system was measured with the stray light signal of the dilute colloidal silica suspension (LUDOX^®^).

Singlet molecular oxygen was detected directly during ^1^O_2_ luminescence spectrum recording in the range from 1200 nm to 1350 nm for C_60_, PyC_60_ and C_60_–CoTTP/PyC_60_–CoTTP systems in toluene. The PyC_60_ luminescence signal of singlet oxygen was measured and compared with the similar C_60_ signal for which $\Phi_{1_{O_{2}}}^{\Delta }$ value in toluene is known [51]. The PyC_60_ $\Phi_{1_{O_{2}}}^{\Delta }$ value was calculated using the equation:

(1) $$\Phi_{1_{O_{2}}}^{\Delta }=\frac{A_{std}S_{PyC_{60}}\tau_{std}}{A_{PyC_{60}}S_{std}\tau_{PyC_{60}}}\Phi_{std}^{\Delta }$$

where *S* is the area of singlet oxygen emitted spectrum, *A* is the absorbance of the solution at the excitation wavelength and *τ* is the singlet oxygen lifetime of PyC_60_ or C_60_. The symbol reported as subscript (std) refers to the C_60_ standard sample.

### 3.5. Femtosecond Laser Photolysis Setup

Femtosecond transient absorption spectra were measured at 20 °C on a pump and light supercontinuum probe setup with a 3.3 fs to 1 ps delay step and a 1 nm wavelength step. The spectra were subjected to correction procedure [64,65,66]. The details of the transient spectra measurement were described in Supplementary Materials. The analysis of the characteristic times (τ) of the transient absorption spectra was carried out using the kinetic simulation based on the singular value decomposition (SVD) of the obtained data matrix. This approach involves the consideration of the entire array of the experimental values Δ*A*(λ, t). Exponential processing of the obtained data at the wavelength of the excited state is used to determine the lifetime of the excited state.

### 3.6. DFT Calculations

The DFT calculations were carried out for (PyC_60_)_2_CoTTP and (C_60_)_2_CoTTP using the Gaussian 16, Revision C.01 program package [67]. The B3LYP functional [68] and the def2-SVP [69] basis set on all atoms (the def2-ECP is automatically assigned to cobalt atoms) were used for the geometric optimization of the triads. Influence of intramolecular dispersion interactions on molecular structure was explored using the dispersion corrections introduced by S. Grimme (D3) [70]. The geometric optimization of the triads was carried out without limitation in symmetry. Harmonic vibrational frequencies were calculated at the same theory level in order to characterize the stationary points as true minima, representing the equilibrium structures on the potential energy surfaces. ChemCraft 1.8 [71] (the graphical program for visualization of quantum chemistry computations, https://chemcraftprog.com) was used for analyses of results and molecular graphics.

The bonding energy (*E_bonding_*) was calculated as follows: *E*_bonding_ = |*E*_triad_ − (*E*_CoTTP_ + *E*_C60/PyC60_)|.

## 4. Conclusions

(5,10,15,20-tetra(4-methylphenyl)porphinato)cobalt(II) (CoTTP) in toluene bonds fullerene (C_60_) and 1-N-methyl-2-(pyridin-4-yl)-3,4-fullero[60]pyrrolidine (PyC_60_) in the two-step two-ways processes end in the noncovalent 1: 2 π-π complex and coordination complex, respectively. The bonding constants are (3.47 ± 0.69) ×10^9^ M^−2^ and (1.47 ± 0.28) ×10^10^ M^−2^, respectively, according to the methods of electron absorption spectroscopy. The fluorescence titration gives the close values of the constants with an approximately 1.3 times higher standard deviation. ^1^H NMR, IR, thermogravimetric analysis and cyclic voltammetry studies of CoTTP, fullerenes and their complexes provide very good support in favor of the effective ground-state complexation between fullerenes and CoTTP. The quenching of the PyC_60_/C_60_ fluorescence and the decreasing intensity of the singlet molecular oxygen phosphorescence parallel to the gradual increase in the CoTTP additions during the steady-state emission experiments have confirmed the photoinduced electron transfer (PET) in the triads, (PyC_60_)_2_CoTTP/(C_60_)_2_CoTTP. The PyC_60_/C_60_ fluorescence lifetime and the exited-state characteristics were obtained using fluorescence and femtosecond transient absorption. DFT calculations used to predict the localization of the frontier orbitals and to determine the HOMO/LUMO energy levels have also confirmed the PET from the singlet excited CoTTP to the fullerene moiety in the triads. These results are of particular importance in the development of the porphyrin-fullerene systems, having potential for energy-harvesting applications.

## Data Availability

Not applicable.

## Supplementary Materials

The following supporting information can be downloaded at: https://www.mdpi.com/article/10.3390/molecules27248900/s1, Figure S1: The transformation of UV–vis spectrum of the CoTTP–5.76 × 10^−5^ M PyC_60_ mixture in toluene at 298 K during 500 s; Figure S2: The plots of log*I* vs. log*C*_PyC60_ at the first and the second stage for the reaction of CoTTP with PyC_60_; Table S1: The rate constants of the (PyC_60_)CoTTP reaction with PyC_60_ in toluene at 298 K; Figure S3: The plot of log*k*_obs_ vs. log*C*_PyC60_ for the reaction of (PyC_60_)CoTTP with PyC_60_ in toluene at 298 K; Figure S4: The UV–vis spectrum transformations during the titration of CoTTP with C_60_ (*C*_C60_ = 0 ÷ 9.0 × 10^−5^ M) in toluene at τ = 0 (a) and τ = ∞ (b). Insets: the plots of log*I* vs. log*C*_C60_; Figure S5: The Benesi–Hildebrand plots for (PyC_60_)_2_CoTTP (a) and (C_60_)_2_CoTTP (b); Table S2: The time-resolved fluorescence spectroscopy results for C_60_, PyC_60_, C_60_–CoTTP and PyC_60_–CoTTP systems; Table S3: The phosphorescence decay of singlet oxygen formed by sensitization with PyC_60_ and the PyC_60_–CoTTP system; Table S4: The IR bands of fullerenes in the (PyC_60_)_2_CoTTP and (C_60_)_2_CoTTP triads; Figure S6: The ^1^H NMR spectra of CoTTP in CDCl_3_; Figure S7: The TG and DTG curves for the CoTTP (a), C_60_ (b) and PyC_60_ (c) powder from 25 °C to 920 °C, with the heating rate of 10 °C/min; Table S5: The peak potentials for CoTTP, C_60_/PyC_60_ and triad based on them in CH_2_Cl_2_ containing 0.1 M (n-Bu)_4_NClO_4_; Figure S8: The values of the (PyC_60_)_2_CoTTP (a) and (C_60_)_2_CoTTP (b) coordination center bond lengths; Figure S9: The Mulliken charge values of the atoms forming the coordination center of CoTTP (a), (PyC_60_)_2_CoTTP (b) and (C_60_)_2_CoTTP (c); Figure S10: The femtosecond transient absorption spectra registered at various time delays for CoTTP in toluene following the 435 nm laser excitation; Figure S11: The transient absorption decays at 443 nm at early time (0–1 ps) (a) and (1–500 ps) (b) in toluene recorded for CoTTP (τ_exc_ = 435 nm), the green line is monoexponential fit to the decay profile; Figure S12: The MALDI-TOF spectrum of CoTTP; thermodynamics and kinetics; femtosecond laser photolysis setup.
