# Supplementary material for: Donor–Acceptor Complexes of (5,10,15,20-Tetra(4-methylphenyl)porphyrinato)cobalt(II) with Fullerenes C60: Self-Assembly, Spectral, Electrochemical and Photophysical Properties
Source: Molecules. 2022 Dec 14;27(24):8900. doi: 10.3390/molecules27248900 (PMC9783012; doi:10.3390/molecules27248900)
Supplement: Supplementary file 1 [file molecules-27-08900-s001.zip › molecules-2087143-supplementary.pdf]

Supporting information

DONOR-ACCEPTOR COMPLEXES OF (5,10,15,20-TETRA(4-METHYLPHENYL)PORPHYRINATO)COBALT(II) WITH FULLERENES C_{60}: SELF-ASSEMBLY, SPECTRAL, ELECTROCHEMICAL AND PHOTOPHYSICAL PROPERTIES

Nataliya G.Bichan[1]*, Ekaterina N. Ovchenkova[1], Varvara A. Mozgova[1], Alexander A. Ksenofontov[1], Nadezhda O. Kudryakova[1], Ivan V. Shelaev[2], Fedor E. Gostev[2], Tatyana N. Lomova[1]

[1]G.A. Krestov Institute of Solution Chemistry of the Russian Academy of Sciences, Akademicheskaya Str. 1, Ivanovo, Russia

[2]N.N. Semenov Federal Research Center for Chemical Physics Russian Academy of Sciences, Kosigin Str. 4, Moscow, Russia

*Corresponding author: bng@isc-ras.ru

Figure S1. The transformation of UV-vis spectrum of the CoTTP – 5.76 × 10^{-5} M PyC_{60} mixture in toluene at 298 K during 500 seconds. 3

Figure S2. The plots of log$I$ *vs* log$C_{PyC_{60}}$ at the first and the second stage for reaction of CoTTP with PyC_{60}........ 3

Table S1. The rate constants of the (PyC_{60})CoTTP reaction with PyC_{60} in toluene at 298 K... 3

Figure S3. The plot of logk_{obs} *vs* logC_{PyC60} for the reaction of (PyC_{60})CoTTP with PyC_{60} in toluene at 298 K. 4

Figure S4. The UV-vis spectrum transformations during the titration of CoTTP with C_{60} (C_{C60} = 0 ÷ 9.0 × 10^{-5} M) in toluene at τ = 0 (*a*) and τ = ∞ (*b*). Insets: the plots of log$I$ *vs* logC_{C60}..... 4

Figure S5. The Benesi–Hildebrand plots for $(PyC_{60})_2$CoTTP (*a*) and $(C_{60})_2$CoTTP (*b*). 4

Table S2. The time-resolved fluorescence spectroscopy results for C_{60}, PyC_{60}, C_{60} – CoTTP and PyC_{60} – CoTTP systems. 5

Table S3. The phosphorescence decay of singlet oxygen formed by sensitization with PyC_{60} and the PyC_{60} – CoTTP system........ 11

Table S4. The IR bands of fullerenes in the $(PyC_{60})_2$CoTTP and $(C_{60})_2$CoTTP triads........ 13

Figure S6. The ^{1}H NMR spectra of CoTTP in $CDCl_3$. 13

Figure S7. The TG and DTG curves for the CoTTP (*a*), C_{60} (*b*), and PyC_{60} (*c*) powder from 25 °C to 920 °C, the heating rate 10 °C/min. 14

Table S5. The peak potentials for CoTTP, C_{60}/PyC_{60} and triad based on them in CH_2Cl_2 containing 0.1 M $(n\text{-}Bu)_4NClO_4$........ 14

Figure S8. The values of the $(PyC_{60})_2$CoTTP (*a*) and $(C_{60})_2$CoTTP (*b*) coordination center bond lengths. 14

Figure S9. The Mulliken charge values of the atoms forming the coordination centre of CoTTP (a), $(PyC_{60})_2$CoTTP (b), and $(C_{60})_2$CoTTP (c) coordination centre........ 15

Figure S10. The femtosecond transient absorption spectra registered at various time delays for CoTTP in toluene following the 435 nm laser excitation. 15

Figure S11. The transient absorption decays at 443 nm at early time (0 – 1 ps) (*a*) and (1 – 500 ps) (*b*) in toluene recorded for CoTTP (λ_{exc} = 435 nm), the green line is monoexponential fit to the decay profile. 15

Figure S12. The MALDI-TOF spectrum of CoTTP. 16

Thermodynamics and kinetics 16

Femtosecond laser photolysis setup 17

Figure S1. The transformation of UV-vis spectrum of the CoTTP – 5.76×10^{-5} M PyC_{60} mixture in toluene at 298 K during 500 seconds.

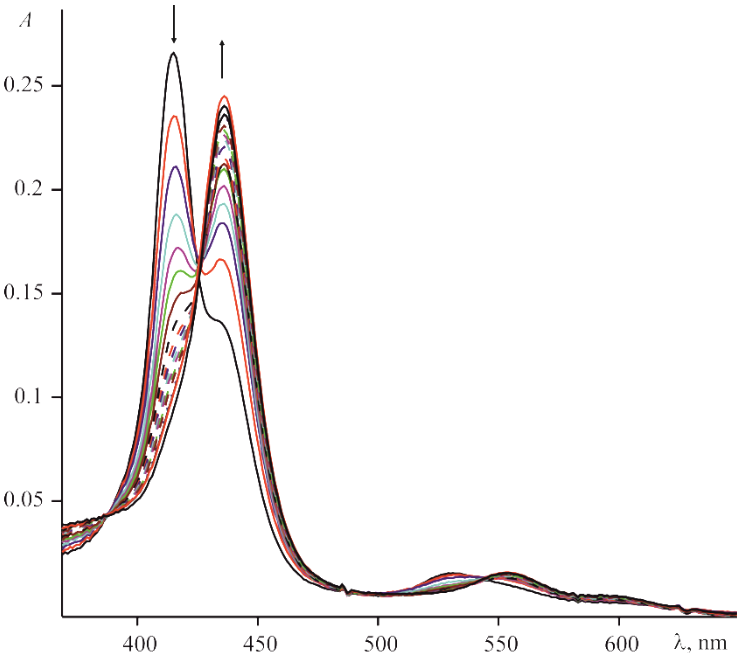

Figure S2. The plots of log*I* *vs* log$C_{PyC_{60}}$ at the first and the second stage for reaction of CoTTP with PyC_{60}.

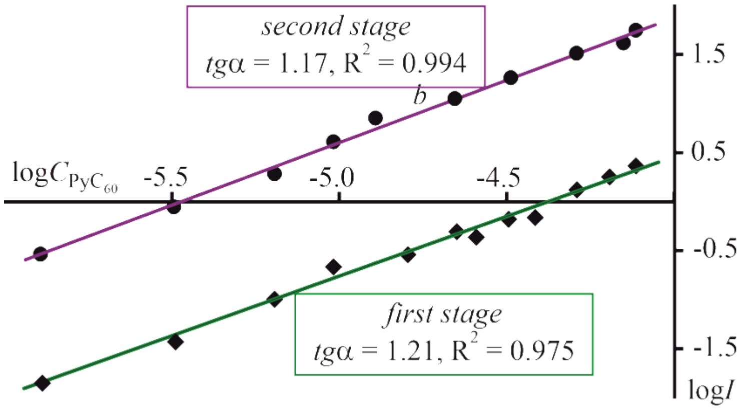

Table S1. The rate constants of the (PyC_{60})CoTTP reaction with PyC_{60} in toluene at 298 K.

$C_{PyC60} \times 10^5$, M	$(k_{2obs} \pm \delta k_{2obs}) \times 10^2$, s^{-1}
1.92	1.73±0.08
2.24	1.85±0.17
2.56	2.04±0.12
3.20	2.22±0.14
3.84	2.65±0.25
4.48	3.19±0.18
5.12	3.77±0.36
5.76	3.92±0.15
7.04	4.24±0.24

Figure S3. The plot of logk_{obs} *vs* logC_{PyC60} for the reaction of (PyC$_{60}$)CoTTP with PyC$_{60}$ in toluene at 298 K.

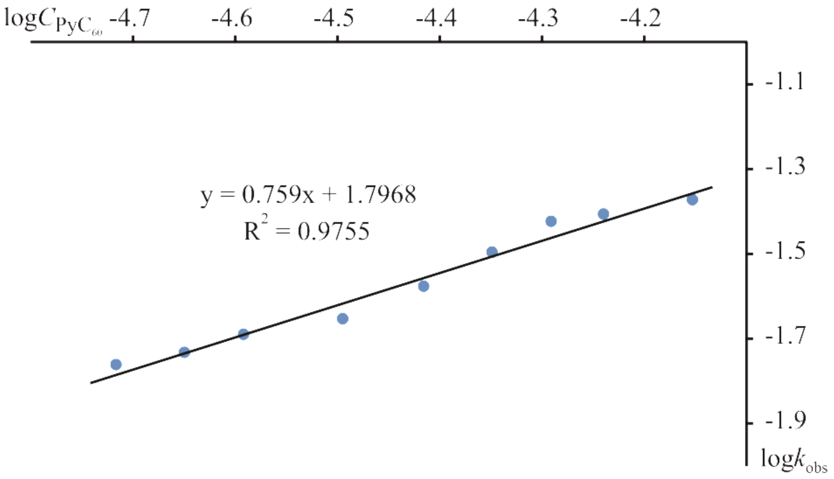

Figure S4. The UV-vis spectrum transformations during the titration of CoTTP with C$_{60}$ (C_{C60} = 0 ÷ 9.0 × 10^{-5} M) in toluene at τ = 0 (*a*) and τ = ∞ (*b*). Insets: the plots of log*I* *vs* logC_{C60}.

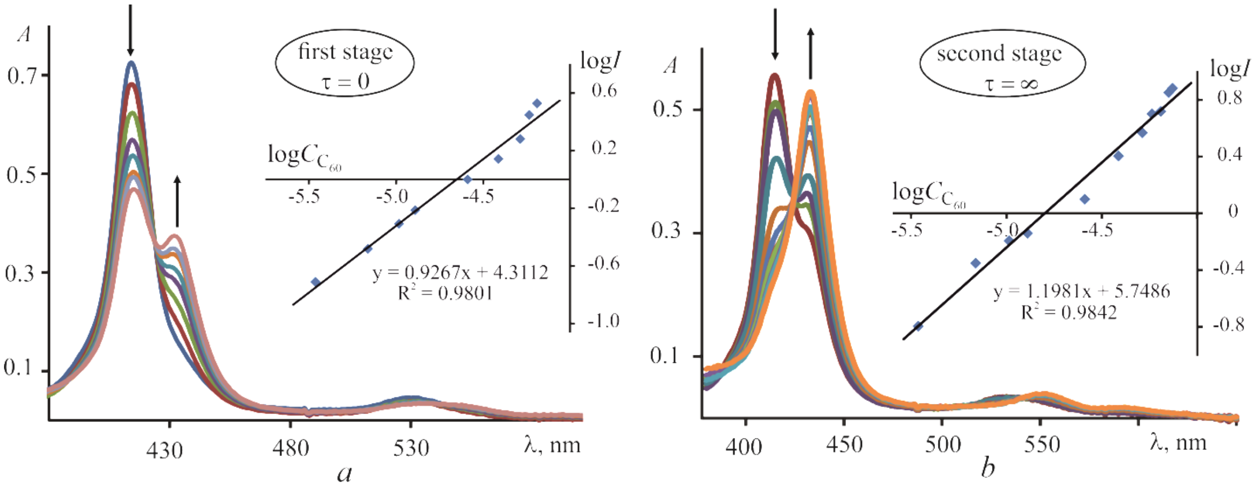

Figure S5. The Benesi–Hildebrand plots for (PyC$_{60}$)$_2$CoTTP (*a*) and (C$_{60}$)$_2$CoTTP (*b*).

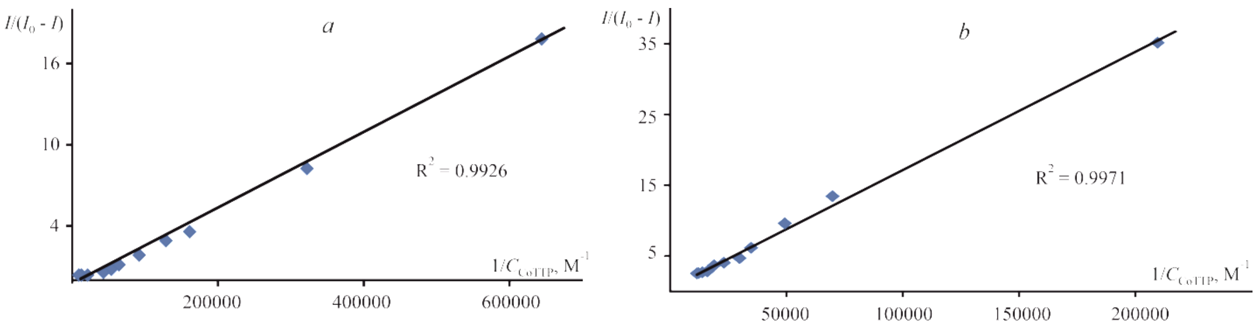

Table S2. The time-resolved fluorescence spectroscopy results for C_{60}, PyC_{60}, C_{60} – CoTTP and PyC_{60} – CoTTP systems.

C_{60} (9.1×10^{-5} M)

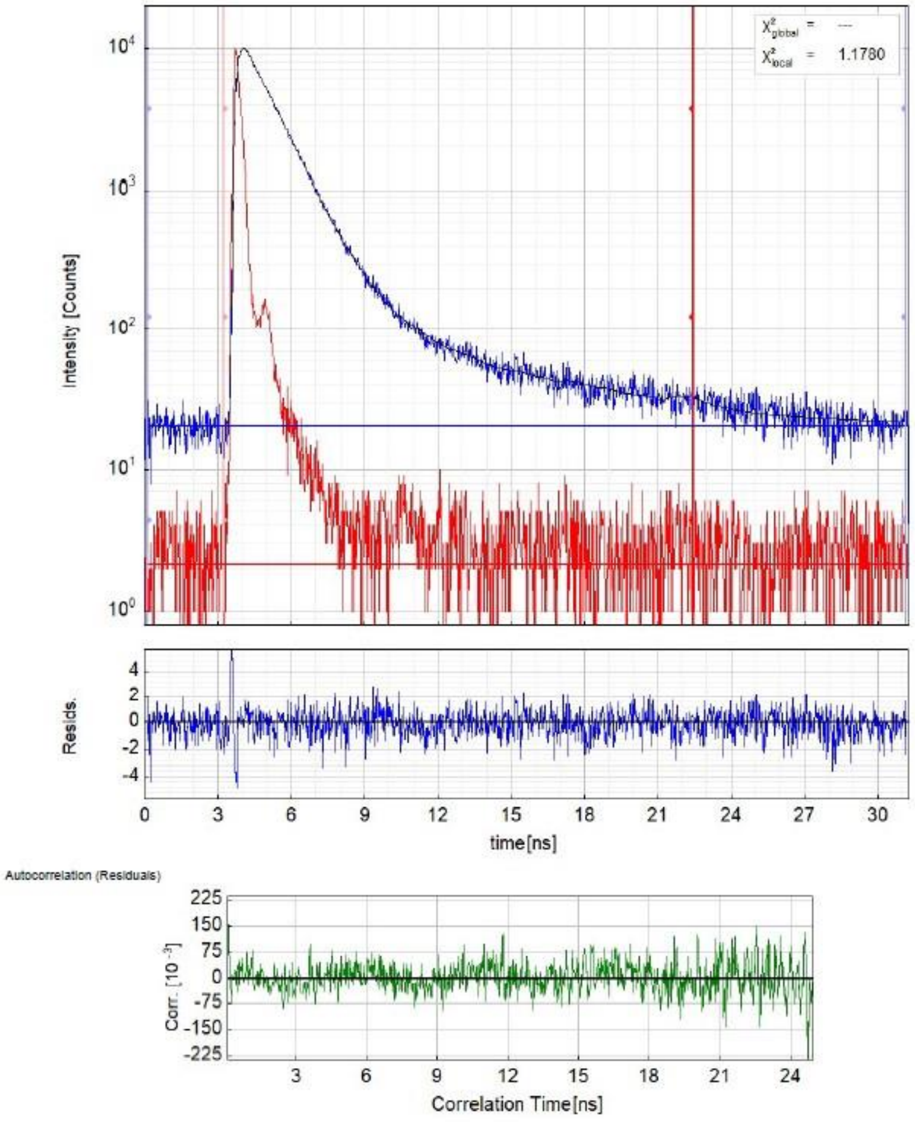

Parameter	Value	Δ	δ
A_1[kCnts/Chnl]	0.174	±0.016	9.0%
$τ_1$[ns]	5.74	±0.32	5.5%
I_1[kCnts]	39.9	±1.2	3.0%
A_2[kCnts/Chnl]	14.646	±0.035	0.2%
$τ_2$[ns]	1.1111	±0.0052	0.5%
I_2[kCnts]	650.9	±2.0	0.3%
$Bkgr_{Dec}$[kCnts]	0.0204	±0.0003	1.1%
$Bkgr_{IRF}$[Cnts/Chnl]	2.159	±0.063	2.9%
$Shift_{IRF}$[ps]	-44.6	±1.9	4.2%
$τ_{AvInt}$[ns]	1.378	±0.017	1.2%

PyC$_{60}$ (6.6 ×10^{-5} M)

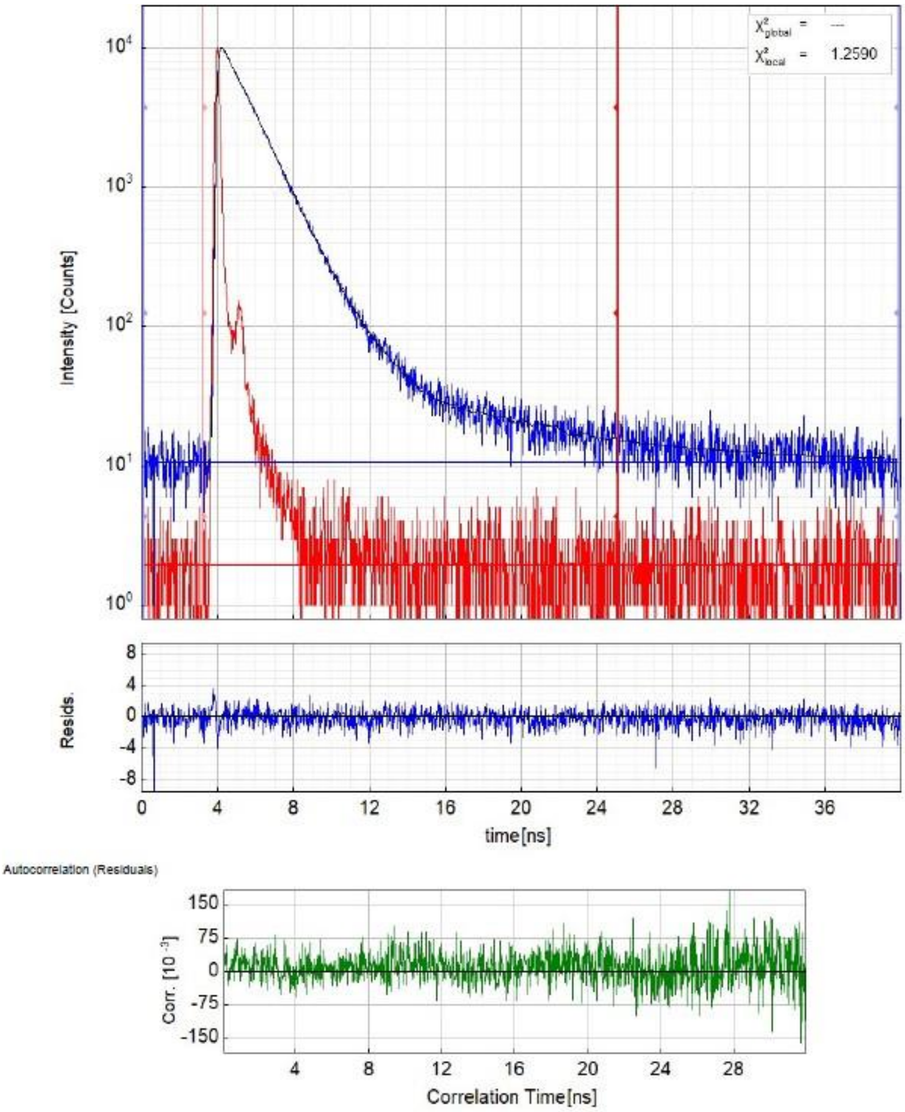

Parameter	Value	Δ	δ
A_1[kCnts/Chnl]	13.156	±0.031	0.2%
τ_1[ns]	1.4326	±0.0043	0.3%
I_1[kCnts]	753.9	±1.7	0.2%
A_2[kCnts/Chnl]	0.0588	±0.0052	8.8%
τ_2[ns]	8.33	±0.47	5.6%
I_2[kCnts]	19.57	±0.91	4.6%
$Bkgr_{Dec}$[kCnts]	0.0106	±0.0003	2.4%
$Bkgr_{IRF}$[Cnts/Chnl]	1.950	±0.062	3.1%
$Shift_{IRF}$[ps]	11.3	±1.4	12%
τ_{AvInt}[ns]	1.607	±0.023	1.4%

C_{60} (9.1×10^{-5} M) + CoTTP (4.8×10^{-6} M)

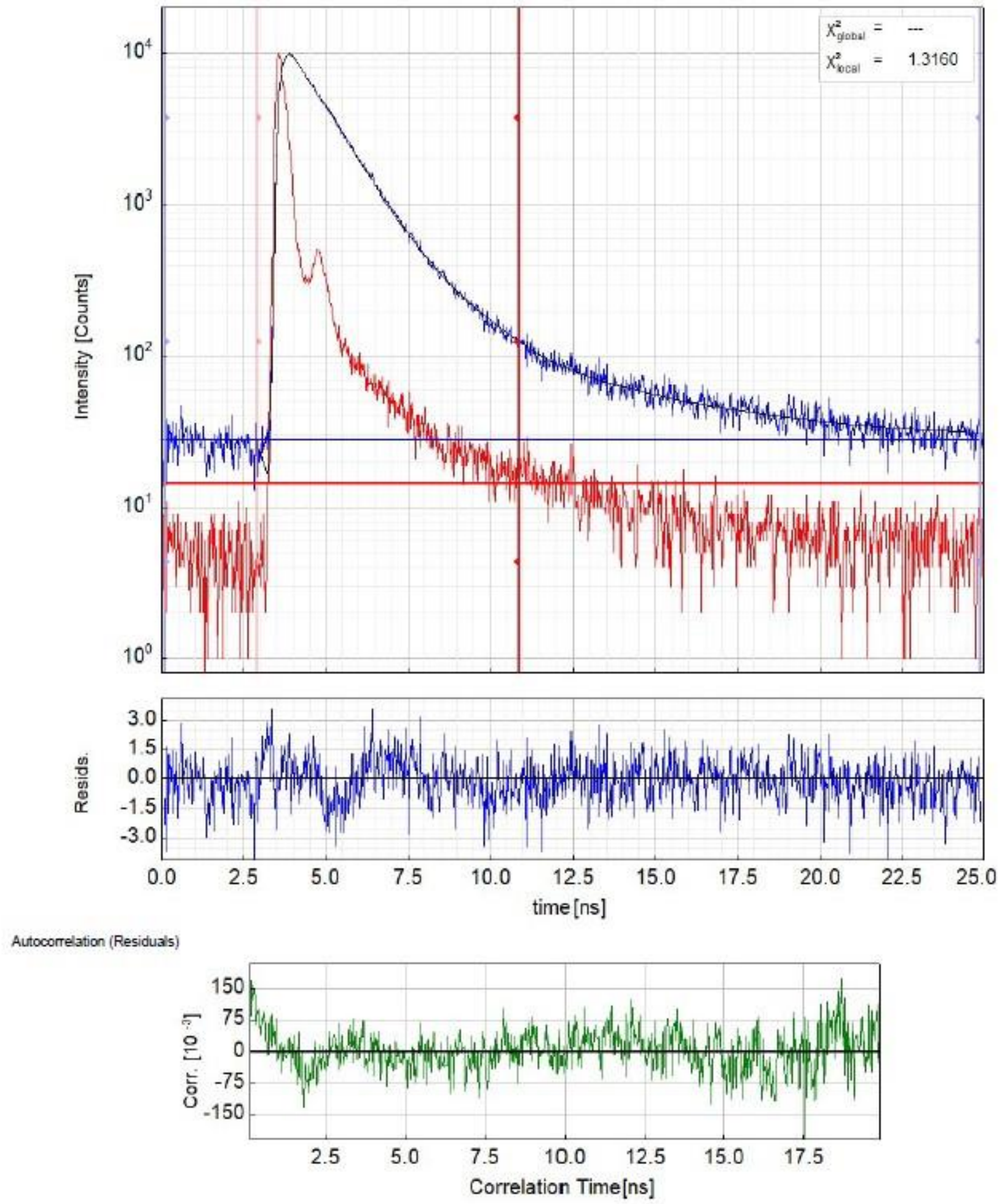

Parameter	Value	Δ	δ
A_1[kCnts/Chnl]	16.790	±0.035	0.2%
τ_1[ns]	0.9530	±0.0067	0.7%
I_1[kCnts]	640.1	±4.7	0.7%
A_2[kCnts/Chnl]	0.294	±0.048	16%
τ_2[ns]	4.73	±0.29	6.0%
I_2[kCnts]	55.4	±6.0	11%
$Bkgr_{Dec}$[kCnts]	0.0278	±0.0007	2.3%
$Bkgr_{IRF}$[Cnts/Chnl]	14.5	±1.3	8.5%
$Shift_{IRF}$[ps]	-8.66	±0.42	4.7%
τ_{AvInt}[ns]	1.254	±0.015	1.1%

C_{60} (9.1 ×10^{-5} M) + CoTTP (2.4 ×10^{-5} M)

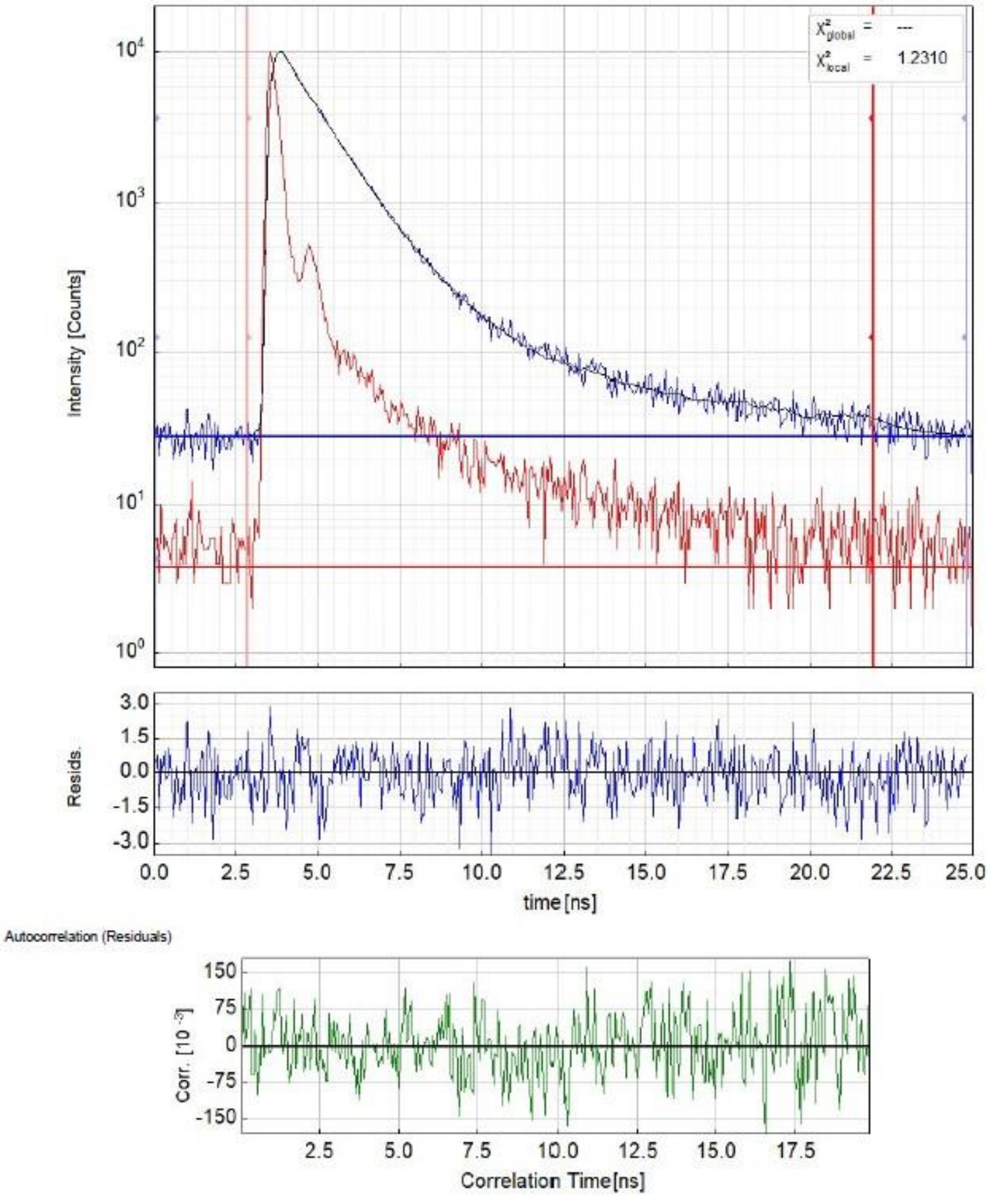

Parameter	Value	Δ	δ
A_1[kCnts/Chnl]	8.5	±2.6	30%
τ_1[ns]	0.66	±0.12	18%
I_1[kCnts]	111	±52	47%
A_2[kCnts/Chnl]	10.1	±3.0	29%
τ_2[ns]	1.204	±0.069	5.7%
I_2[kCnts]	243	±53	22%
$Bkgr_{Dec}$[kCnts]	0.0285	±0.0004	1.3%
$Bkgr_{IRF}$[Cnts/Chnl]	3.79	±0.16	4.1%
$Shift_{IRF}$[ps]	-2.6	±1.2	46%
τ_{AvInt}[ns]	1.0306	±0.0047	0.5%

PyC$_{60}$ (6.6 ×10^{-5} M) + CoTTP (2.4 ×10^{-5} M)

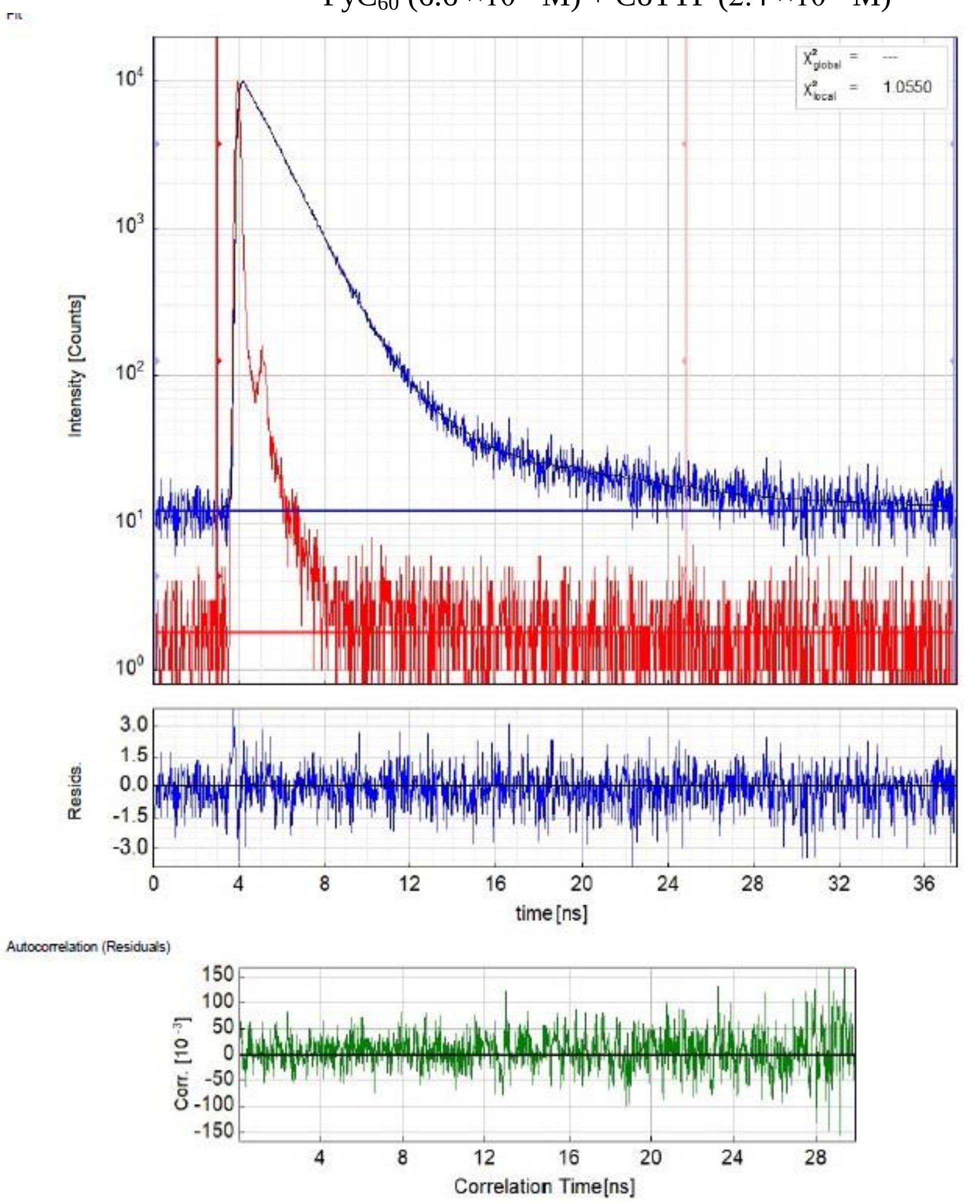

Parameter	Value	Δ	δ
A_1[kCnts/Chnl]	12.949	±0.031	0.2%
$τ_1$[ns]	1.4331	±0.0028	0.2%
I_1[kCnts]	742.3	±1.4	0.2%
A_2[kCnts/Chnl]	0.0669	±0.0068	10%
$τ_2$[ns]	7.79	±0.59	7.5%
I_2[kCnts]	20.84	±1.00	4.8%
$Bkgr_{Dec}$[kCnts]	0.0122	±0.0002	1.5%
$Bkgr_{IRF}$[Cnts/Chnl]	1.77	±0.12	6.3%
$Shift_{IRF}$[ps]	10.63	±0.89	8.4%
$τ_{AvInt}$[ns]	1.607	±0.020	1.2%

PyC$_{60}$ (6.6 ×10^{-5} M) + CoTTP (4.8 ×10^{-5} M)

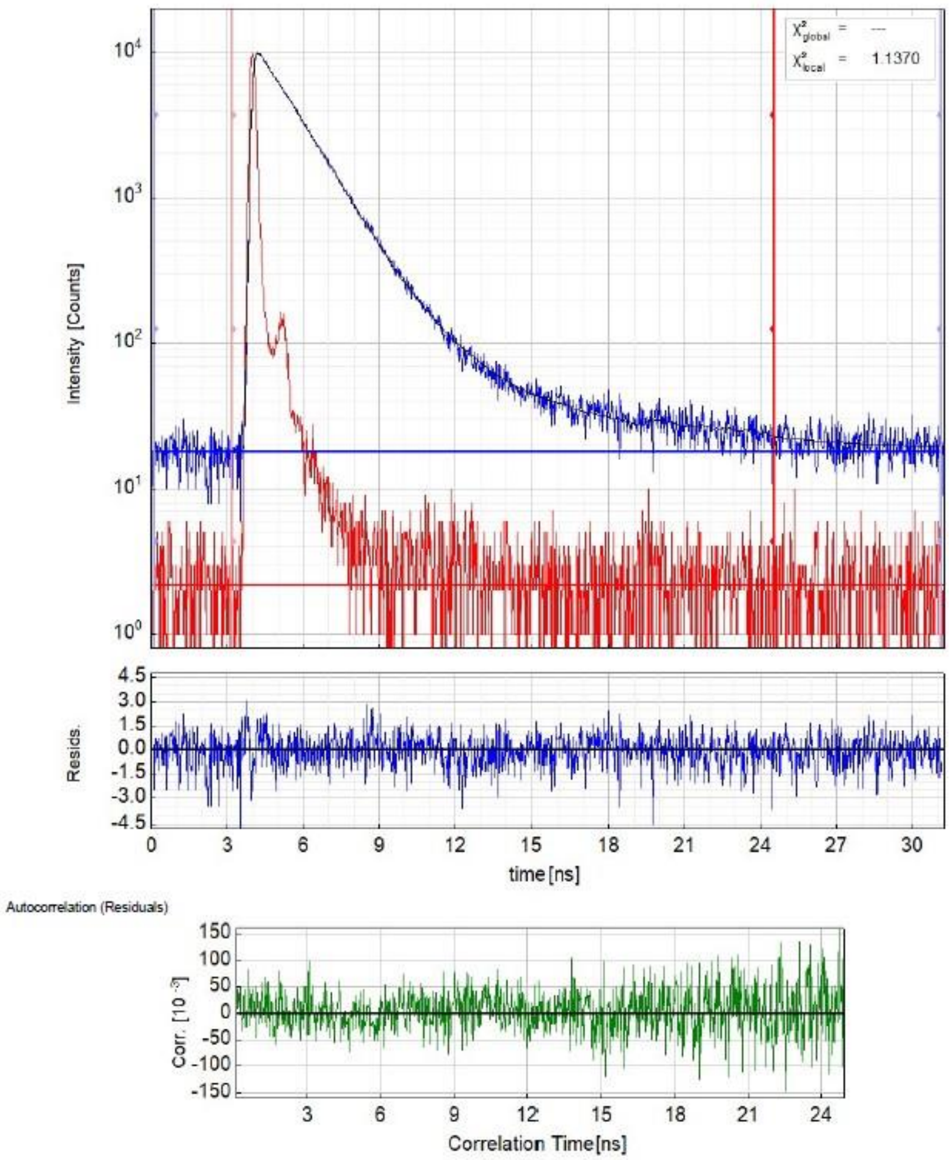

Parameter	Value	Δ	δ
A_1[kCnts/Chnl]	12.897	±0.029	0.2%
τ_1[ns]	1.4241	±0.0033	0.2%
I_1[kCnts]	734.6	±1.6	0.2%
A_2[kCnts/Chnl]	0.0924	±0.0070	7.5%
τ_2[ns]	6.67	±0.41	6.1%
I_2[kCnts]	24.7	±1.3	5.0%
$Bkgr_{Dec}$[kCnts]	0.0179	±0.0005	2.7%
$Bkgr_{IRF}$[Cnts/Chnl]	2.17	±0.12	5.1%
$Shift_{IRF}$[ps]	-2.29	±0.80	35%
τ_{AvInt}[ns]	1.595	±0.012	0.7%

Table S3. The phosphorescence decay of singlet oxygen formed by sensitization with PyC_{60} and the PyC_{60} – CoTTP system.

PyC_{60} (7.48×10^{-5} M)

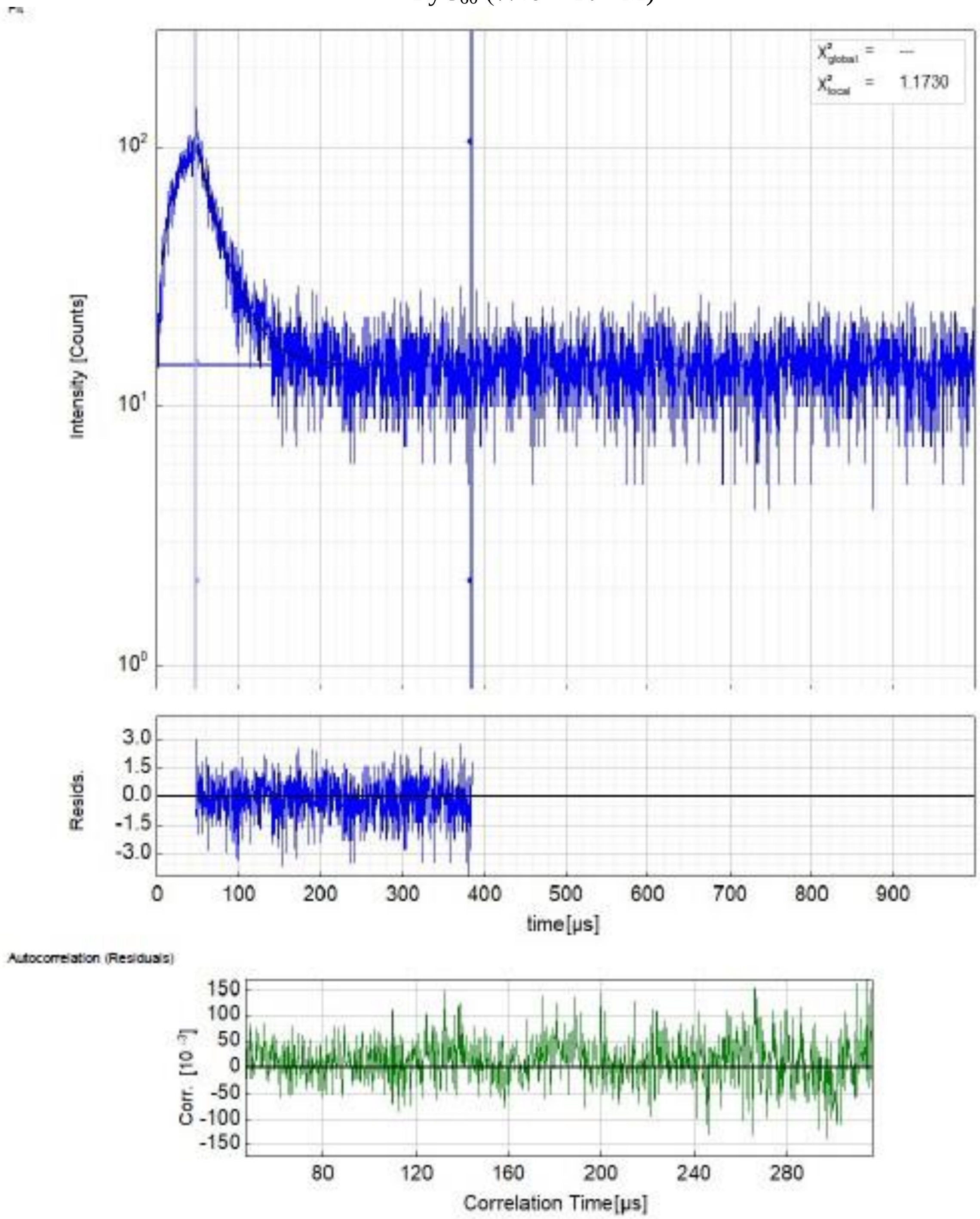

Parameter	Value	Δ	δ
A_1[kCnts/Chnl]	0.0926	±0.0025	2.6%
τ_1[ns]	28 500	±1 100	3.6%
I_1[kCnts]	8.25	±0.13	1.5%
$Bkgr_{Dec}$[kCnts]	0.0144	±0.0002	1.2%
τ_{AvInt}[ns]	28 500	±1 100	3.6%

PyC$_{60}$ (6.19 × 10^{-5} M) - CoTTP (5.57 × 10^{-6} M)

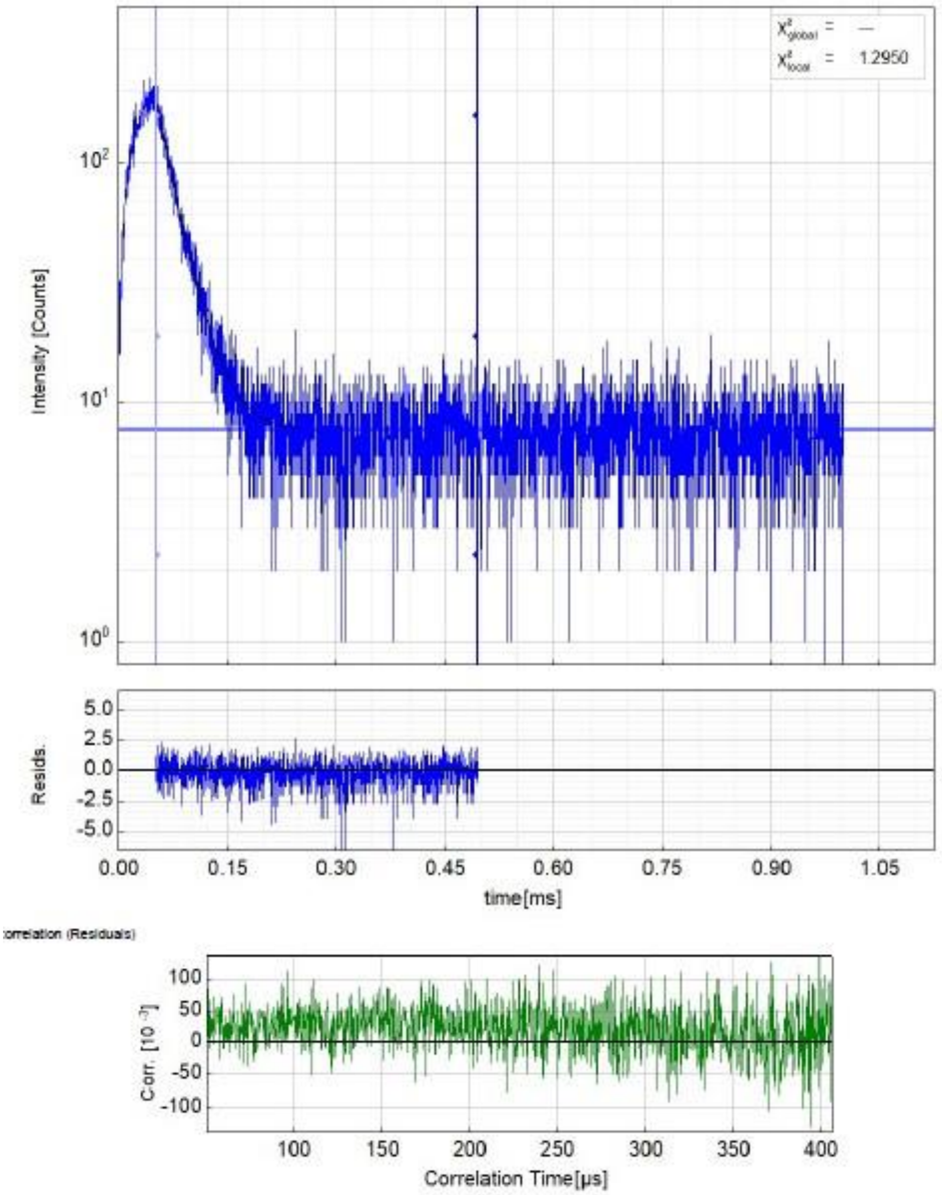

Parameter	Value	Δ	δ
A_1[kCnts/Chnl]	0.1798	±0.0022	1.2%
τ_1[ns]	28 190	±260	0.9%
I_1[kCnts]	15.83	±0.15	0.9%
$Bkgr_{Dec}$[kCnts]	0.0077	±0.0001	0.9%
τ_{AvInt}[ns]	28 190	±260	0.9%

Table S4. The IR bands of fullerenes in the $(PyC_{60})_2CoTTP$ and $(C_{60})_2CoTTP$ triads.

IR bands	
**C_{60}**	**$(C_{60})_2CoTTP$**
the signals of the C_{60} skeleton	
1428<br>1183<br>577<br>527	1428<br>1182<br>576<br>527
**PyC_{60}**	**$(PyC_{60})_2CoTTP$**
the signals of the C_{60} skeleton	
1430<br>1179<br>574<br>527	1428<br><br>574<br>527
the signals of the pyridyl/pyrrolidinyl fragments	
1268	1281
1246	1245
707	709
664	670
635	635
598	598
574	583
553	552
527	541
504	505
486	486
479	479
413	413

Figure S6. The ^1H NMR spectra of CoTTP in $CDCl_3$.

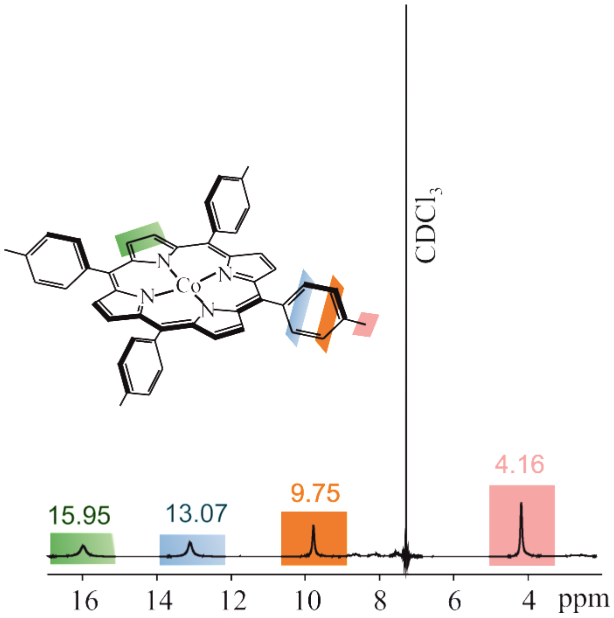

Figure S7. The TG and DTG curves for the CoTTP (*a*), C_{60} (*b*), and PyC_{60} (*c*) powder from 25 °C to 920 °C, the heating rate 10 °C/min.

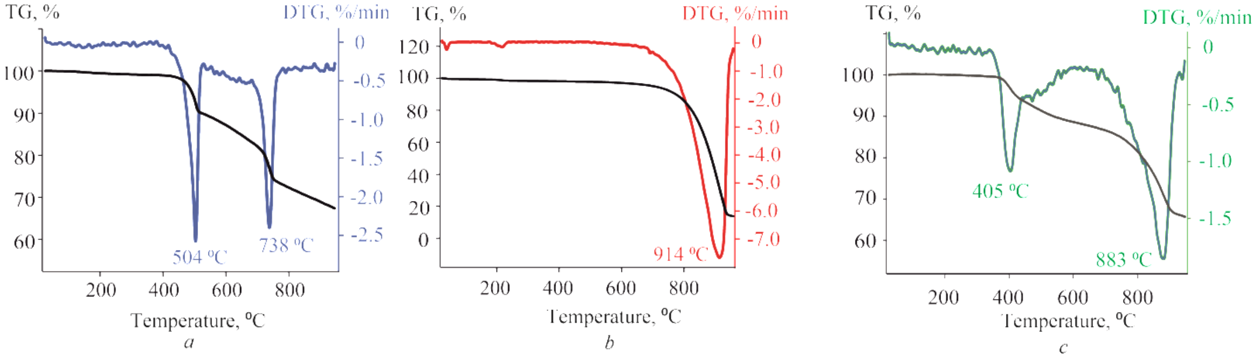

Table S5. The peak potentials for CoTTP, C_{60}/PyC_{60} and triad based on them in CH_2Cl_2 containing 0.1 M $(n\text{-}Bu)_4NClO_4$

Compound	An oxidation peak, V					A reduction peak, V				
CoTTP	1.22	0.98				0.84	0.51			
PyC_{60}			-0.57	-1.04	-1.55			-0.78	-1.10	-1.62
C_{60}			-0.55	-1.18	-1.37			-0.55	-0.98	-1.45
$(PyC_{60})_2$CoTTP	1.36	1.20	-0.61	-1.0	-1.34	1.34	1.07	-0.68	-1.08	-1.45
$(C_{60})_2$CoTTP	1.15	0.87	-0.56	-1.04	-1.38	1.05	0.62	-0.57	-0.94	-1.45

Figure S8. The values of the $(PyC_{60})_2$CoTTP (*a*) and $(C_{60})_2$CoTTP (*b*) coordination center bond lengths.

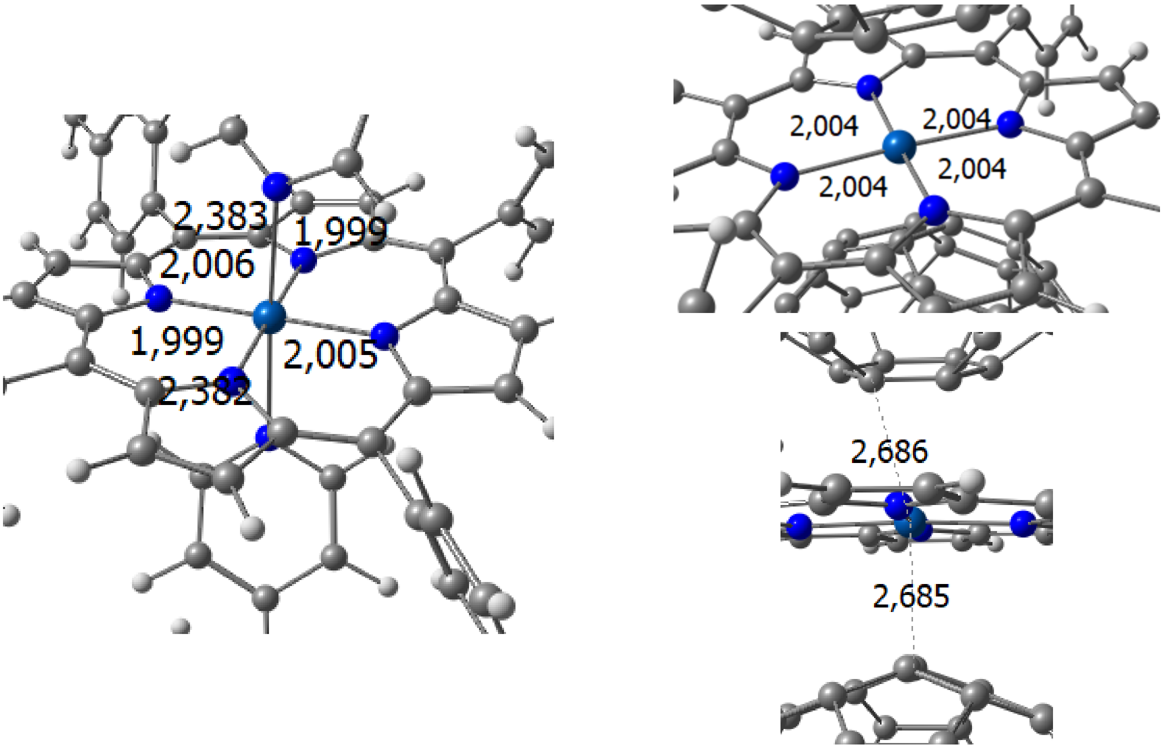

a *b*

Figure S9. The Mulliken charge values of the atoms forming the coordination centre of CoTTP (a), $(PyC_{60})_2$CoTTP (b), and $(C_{60})_2$CoTTP (c) coordination centre.

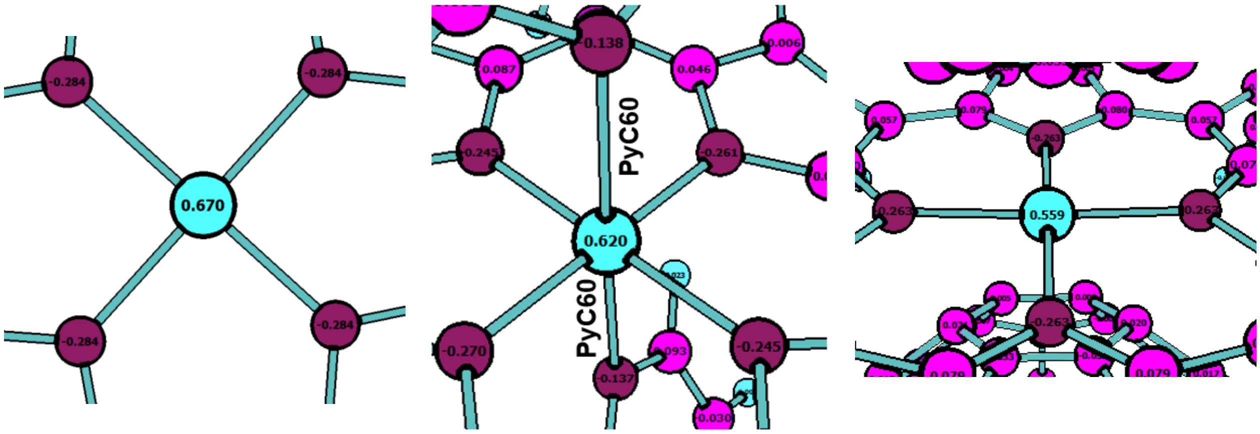

a *b* *c*

Figure S10. The femtosecond transient absorption spectra registered at various time delays for CoTTP in toluene following the 435 nm laser excitation.

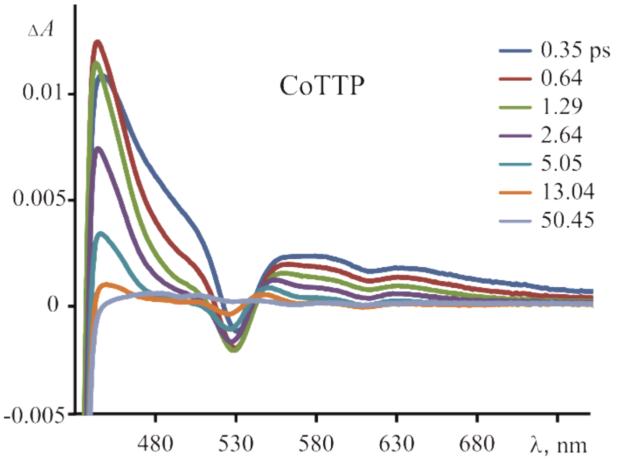

Figure S11. The transient absorption decays at 443 nm at early time (0 – 1 ps) (*a*) and (1 – 500 ps) (*b*) in toluene recorded for CoTTP (λ_{exc} = 435 nm), the green line is monoexponential fit to the decay profile.

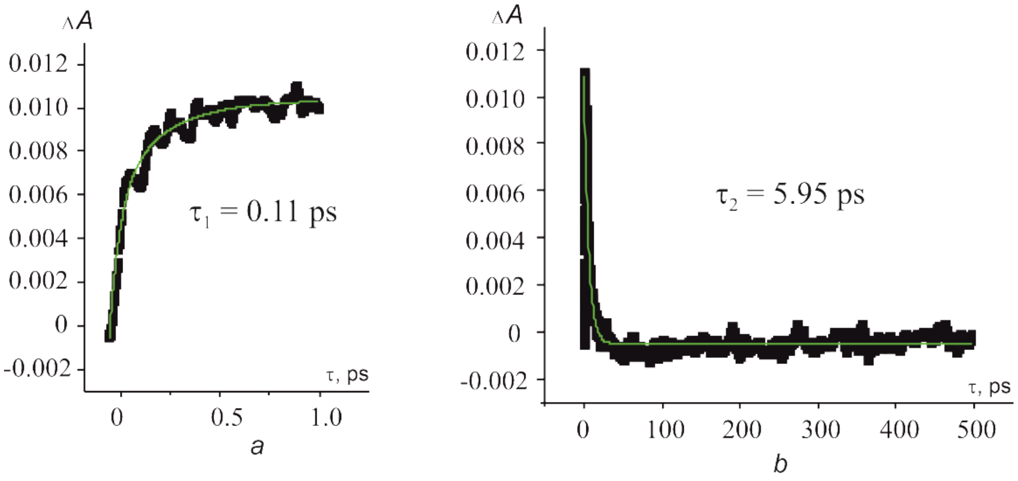

Figure S12. The MALDI-TOF spectrum of CoTTP.

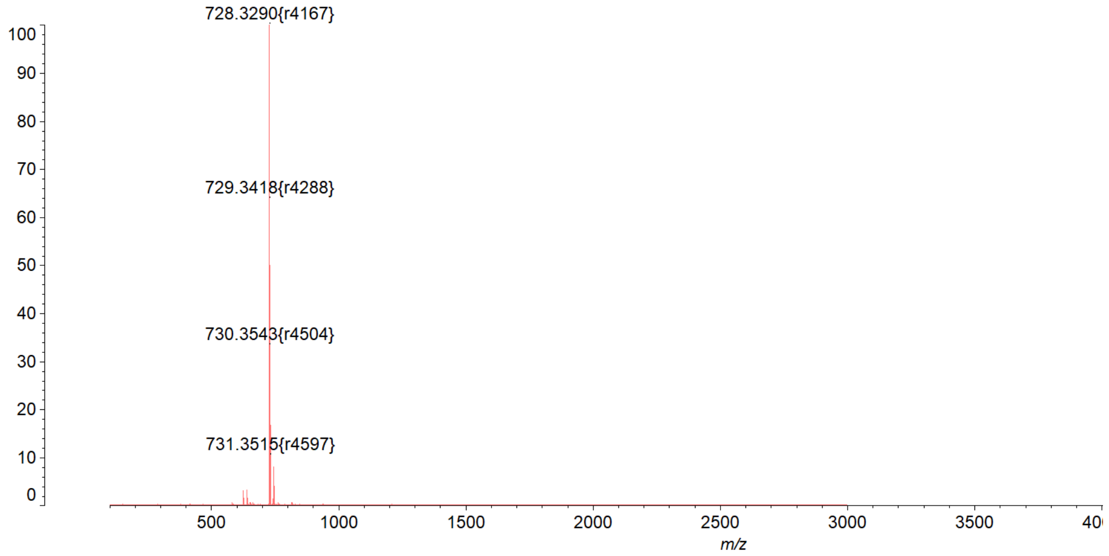

Thermodynamics and kinetics

The solutions with the CoTTP constant concentration (3.8×10^{-6} M) and different concentrations of C_{60} (from 0 to 9.0×10^{-5} M) and PyC_{60} (from 0 to 7.7×10^{-5} M) were prepared. The overall volume of equilibrium mixtures was maintained by using required amounts of the CoTTP solution in toluene, the C_{60}/PyC_{60} solution in toluene, and the pure solvent. Since fullerene absorbs in the UV-vis region, the spectra of the equilibrium mixtures of CoTTP and its complex with C_{60}/PyC_{60} were monitored in a subtraction mode using the spectrum of C_{60}/PyC_{60} of a same concentration as in the stock solution as a zero line.

The constants of the slow equilibriums were calculated by the equation (1) for a three-component equilibrium system:

$$K = \frac{(A_i - A_0)/(A_\infty - A_0)}{1-(A_i - A_0)/(A_\infty - A_0)} \cdot \frac{1}{(C^0_{\mathrm{ful}} - C^0_{\mathrm{CoTTP}} \cdot (A_i - A_0)/(A_\infty - A_0))} \qquad (1)$$

Here, C^0_{ful} and C^0_{CoTTP} is the initial concentrations of C_{60}/PyC_{60} and CoTTP in toluene, respectively; A_0, A_i, and A_∞ is an absorbance at the working wavelength of initial CoTTP, equilibrium mixture at the definite C_{60}/PyC_{60} concentration, and a reaction product. The K values were optimized by the least squares procedure using Microsoft Excel. The relative error in the determination of K did not exceed 25%. The reaction stoichiometry was determined as the slope of the straight line $\log I$ - $f(\log C_{\mathrm{ful}})$ where I is $(A_i - A_0)/(A_\infty - A_i)$.

The kinetics of the slow reaction between the 1: 1 coordination complex, (C_{60})CoTTP /(PyC_{60})CoTTP and the second C_{60}/PyC_{60} molecule in toluene was studied at 298 K by the excess concentrations method. The CoTTP solutions in the freshly distilled toluene were prepared directly before use. To determine the reaction rate constants, the absorbance of the solutions at the constant CoTTP concentration and the increasing C_{60}/PyC_{60} concentration were measured not only immediately after mixing of the reagents when the first fast equilibrium is observed but also over time.

The first order rate constant values, k_{obs}, were calculated using equation (2). Kinetic coefficient, k, was observed using the plot of $\log k_{\mathrm{obs}}$ *vs* $\log C_{\mathrm{ful}}$.

$$k_{obs} = 1/\tau \cdot \ln((A_0 - A_\infty)/(A_\tau - A_\infty)) \qquad (2)$$

Here, A_0, A_τ, A_∞ is the absorbance at the working wavelength for CoTTP, the reaction mixture at the time of τ and a reaction product. A relative error in the determination of k_{obs} did not exceed 10%.

Femtosecond laser photolysis setup

Femtosecond transient absorption measurements were performed using a regeneratively amplified Ti:Sapphire laser system. The output of a Ti:Sapphire oscillator (800 nm, 80 MHz, 30 fs, ‘‘Tsunami’’, Spectra-Physics, USA) was amplified using a regenerative amplifier (‘‘Spitfire Pro’’, Spectra-Physics, USA). The repetition rate of the amplified laser pulses was set at 100 Hz. This frequency is small enough to eliminate permanent bleaching of a sample and corresponds at the same time to the maximum performance of a registration system.

The amplified pulses (800 nm, 100 Hz, 1.5 mJ, 40 fs) were split into two beams. One of the beams was attenuated to 1–2 mJ and focused into a nonlinear optical crystal (a-BBO) to produce the second harmonic radiation, which was used as a pump pulse. The pump pulse had the Gaussian pulse shape centered at the wavelength of 400 nm, the 50 fs duration, and the 100 nJ pulse energy. This energy is optimal for obtaining the signal of sufficient amplitude, and is at the same time small enough to exclude nonlinear processes in a sample.

The second beam was attenuated to 0.8 mJ and directed to non-collinear optical parametric amplifier (‘‘Topas-white’’, Light Conversion, Lithuania) whose radiation (900 nm, 3 5 fs, 10 mJ) was attenuated to 1 mJ and focused into a thin quartz cell with pure H_2O to produce a supercontinuum probe pulse. The supercontinuum probe pulse was attenuated and spectrally filtered from the excess radiation at the wavelength of 900 nm and had the smooth spectrum in the wavelength range of 380–850 nm and the negligible energy (~1 nJ) to exclude its influence on an observed signal.

The pump and probe pulses were delayed relative to each other by the computer-controlled delay line in the range of 0 – 500 ps, with a delay step from 3.3 fs to 1 ps. The pulses were then attenuated, recombined, and focused onto a flow sample cell. The pump and probe light spots had diameters of 300 mm and 120 mm, respectively. The relative polarizations of the pump and probe beams were adjusted to 54.7º (the magic angle).

The experiments were carried out at 293 K. The circulation rate in a flow cell was fast enough to avoid multiple excitations in the sample volume and was 8 mL min^{-1}. Pure dry argon was fed into a flow system to avoid contact of a sample solution with air.

The supercontinuum probe signal out of a sample was dispersed using a polychromator (‘‘Acton SP-300’’, Roper Scientific, USA) and detected using a CCD camera (‘‘Newton’’, Andor, USA). Transient absorption spectral changes $\Delta A(t, \lambda)$ were recorded within the range of 400 - 850 nm. The measured spectra were corrected for group delay dispersion of supercontinuum using the procedure described previously [64, 65]. The zero time delay between pump and probe pulses was determined in control experiments by recording the signal of the non-resonant coherent burst from pure de-aerated toluene.

Particular attention was paid to the ‘‘coherence spike’’ or ‘‘coherent artifact’’ which is observed at initial delays during the overlap of pump and probe pulses and complicates the analysis of the measurement results at delays of < 70 fs. The problem of the resonance signal of a chromophore molecule in a solution for the ‘‘coherent spike’’ time window was analyzed in accordance with the reference [66].
